# Genetic insights revealed ADRB1 as potential target for clear cell renal cell carcinoma

**DOI:** 10.3389/fphar.2026.1838896

**Published:** 2026-06-03

**Authors:** Honghui Zhu, Qi Lin, Zhixian Yu, Xixi Huang

**Affiliations:** Department of Urology, The First Affiliated Hospital of Wenzhou Medical University, Wenzhou, Zhejiang, China

**Keywords:** antihypertensive drug targets, clear cell renal cell carcinoma risk, mendelian randomization, colocalization analysis, genetic variants

## Abstract

**Background:**

Antihypertensive drug targets are associated with various cancers, but their relationship with clear cell renal cell carcinoma (CCRCC) risk remains unclear.

**Methods:**

Summary-data-based Mendelian randomization (SMR) and colocalization analyses were performed. Four antihypertensive drug targets (ACE, ADRB1, ADRB2, and SLC12A3) and CCRCC were included. Patients with CCRCC were identified from two large GWAS databases, including 752,817 and 315,137 individuals (Finnish cohorts), for the discovery and external validation analyses, respectively. Meta-analysis was conducted to integrate the results from both cohorts. Western blotting and prognostic analyses of tumor survival revealed the relationship between ADRB1 and CCRCC.

**Results:**

ADRB1 was associated with CCRCC risk in both the discovery and validation cohorts (odds ratio (OR): 1.097, per standard deviation unit (SD) change in antihypertensive drug target perturbation equivalent to 1 SD unit of decreased blood pressure; 95% confidence interval (95% CI): 1.063–1.132; P-value = 0.016) vs. OR: 1.284; 95% CI: 1.014–1.627; P-value = 0.013). ADRB2 was associated with CCRCC risk in discovery cohort (OR: 1.224; 95% CI: 1.045–1.433; P-value = 0.019). Integrated outcomes demonstrated that both ADRB1 (OR: 1.100; 95% CI: 1.066–1.135; P-value<0.0001) and ADRB2 (OR: 1.313; 95% CI: 1.137–1.517; P-value = 0.0002) were associated with CCRCC risk. Colocalization analyses indicated that ADRB1 (PP4 = 0.996) and ADRB2 (PP4 = 0.895) shared the same region of genetic variation with CCRCC. Furthermore, ADRB1 was highly expressed in CCRCC tumor tissues and was associated with poor tumor survival and prognosis.

**Conclusion:**

ADRB1 was associated with the risk of CCRCC, providing additional perspectives into potential treatment strategies for CCRCC.

## Introduction

Renal cell carcinomas (RCCs) are a heterogeneous group of malignant tumors originating from the renal cortex, each characterized by distinct clinical, morphological, and genetic features (Young et al., 2024). RCC poses a major global health challenge (Jonasch et al., 2021). Among its histological subtypes, clear cell renal cell carcinoma (CCRCC) is the most common, accounting for 70%–80% of all RCC cases (Meng et al., 2023). Unfortunately, patients with CCRCC show limited responsiveness to conventional treatments such as cytotoxic chemotherapy, cytokine therapy, and radiation therapy, and the underlying therapeutic mechanisms remain poorly understood (Zhao and Eyzaguirre, 2019). Furthermore, the lack of effective screening methods for CCRCC underscores the urgent need for improved preventive strategies to reduce its impact (Parikh and Lara, 2018).

Hypertension represents a major gobal public health concern (Carey et al., 2021). To date, more than one billion individuals worldwide are affected by this condition and its associated consequences (Dzau and Hodgkinson, 2024). As the prevalence of hypertension continues to rise, an increasing number of individuals are relying on antihypertensive medications to regulate their blood pressure and maintain it within a healthy range (Benjamin et al., 2019). Considering the widespread use of antihypertensive medications, concerns have arisen regarding the potential adverse effects associated with their long-term use. While studies have demonstrated the relatively short-term safety of certain commonly prescribed antihypertensive drugs, including angiotensin-converting enzyme (ACE) inhibitors, with a median follow-up of 3.5 years, emerging evidence suggests more complex risks (Copland et al., 2021). Frequently used drugs such as ACE inhibitors may contribute to tumor proliferation, migration, and angiogenesis (Sanidas et al., 2020; Hicks et al., 2018). Morever, ADRB1 is highly expressed in renal juxtaglomerular cells and regulates renin secretion (Chen et al., 2026). Dysregulation of adrenergic signaling has been implicated in renal cell proliferation and hypoxia-inducible factor pathways, which are central to CCRCC pathogenesis (Naas et al., 2026). Moreover, studies have suggested a possible significant association between the pharmacological targets of antihypertensive agents and the onset and progression of various cancers, such as breast cancer, hepatocellular carcinoma, prostate cancer, and kidney cancer (Yarmolinsky et al., 2022; Zheng et al., 2024; Wang et al., 2023). While these observations suggest a relationship between antihypertensive drug targets and cancer pathogenesis, a critical question remains unanswered: whether genetically predicted expression of specific antihypertensive drug targets exerts a causal influence on CCRCC risk remains entirely unexplored.

Mendelian randomization (MR) is a robust analytical method used to infer causal relationships between exposures and outcomes by leveraging genetic variants as instrumental variables (IVs) (Sekula et al., 2016). These genetic variants, randomly assigned at conception, act as natural proxies for exposures. This random allocation mimics the randomization process of randomized controlled trials (RCTs), making MR less prone to confounding and reverse causality—limitations often encountered in conventional observational studies (Birney, 2022). Unlike traditional approaches, MR can provide more reliable causal effect estimates without requiring potentially harmful interventions, offering valuable insights into long-term risks and benefits (Zeitoun and El-Sohemy, 2023). In recent years, MR has gained prominence, especially in drug target MR analysis (Burgess et al., 2023). This approach uses genetic variants located within or near genes encoding drug targets to predict the effects of pharmacological interventions on disease outcomes (Evans, 2022). By focusing on variants that influence the expression or function of target genes, MR enables the evaluation of therapeutic interventions, including antagonists, agonists, activators, or inhibitors (Schmidt et al., 2020). Recently, MR has proven particularly useful in studying the effects of antihypertensive drugs. Researchers have employed drug target-related genetic proxies to investigate associations between these medications and disease risk factors, such as psychiatric disorders, fracture, and cancer (Chauquet et al., 2021; Yarmolinsky et al., 2022; Huang et al., 2023). By harnessing the natural randomization inherent in genetic variants, MR serves as a powerful tool for identifying promising therapeutic targets and predicting intervention outcomes, thereby deepening our understanding of disease mechanisms and refining treatment strategies (Evans, 2022).

In this study, a drug target MR approach was employed to elucidate the association between antihypertensive medications and the risk of CCRCC, offering valuable insights into the therapeutic application of these drugs. Moreover, these results may encourage the repurposing of antihypertensive agents as potential preventive strategies against CCRCC, guiding the design of future clinical trials.

## Methods

### Summary-data-based Mendelian randomization analysis

**FIGURE 1:**
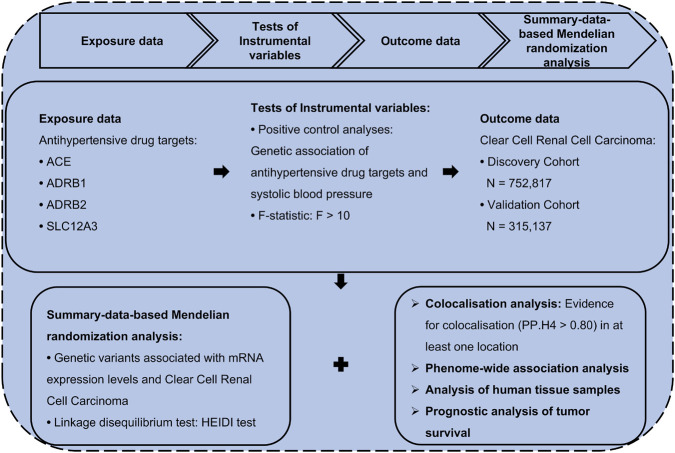
The flow chart of this summary-data-based Mendelian randomization study.

These summary-data-based Mendelian randomization (SMR) analyses were conducted in accordance with the STROBE-MR statement (Skrivankova et al., 2021). The SMR analyses, which integrate expression quantitative trait locus (eQTL) data with summary-level data from genome-wide association studies (GWASs), wereperformed to elucidate the genetic connection between antihypertensive drug targets and CCRCC risk at the gene expression level (Zhu et al., 2016). Moreover, colocalization analysis, grounded in Bayes’ theorem, was used to elucidate the associated between antihypertensive drug targets and CCRCC risk within a ±500 KB window surrounding the gene encoding each independent exposure (Giambartolomei et al., 2014). The study flowchart is illustrated in Figure 1 **.**

### Exposure data

This SMR study ultimately included four targets associated with three antihypertensive drugs. Initially, we identified these targets through the DrugBank (https://www.drugbank.ca) and ChEMBL (https://www.ebi.ac.uk/chembl) databases (Wishart et al., 2018; Zdrazil et al., 2024). Angiotensin-converting enzyme (ACE) inhibitors are primarily used to treat hypertension, heart failure, and acute myocardial infarction (Brouwers et al., 2021). β-1 adrenergic receptor (ADRB1) andβ-2 adrenergic receptor (ADRB2) are recognized as effective targets for β blockers, and are commonly prescribed for various hypertension-related clinical conditions (Messerli et al., 2023). SLC12A3, targeted by thiazide diuretics (NCC; sodium-chloride symporter), serves as a first-line treatment for hypertension and can reduce morbidity outcomes such as cardiovascular events and adverse effects (Reinhart et al., 2023). These drug targets have been confirmed to play substantial roles in blood pressure regulation.

To obtain more effective IVs, common eQTLs (minor allele frequency (MAF) > 1%) were identified from the eQTLGen Consortium database (https://www.eqtlgen.org/) as significant IVs (P < 5 × 10^−8^) associated with ADRB1, ADRB2, and SLC12A3 expression in blood tissue (Supplementary Table S1) (van der Wijst et al., 2020). As no significant eQTLs for ACE were found in aforementioned database, we sourced IVs related to ACE expression from GTEx Consortium Version 8.0 (https://gtexportal.org/), specifically in muscle and skeletal tissues (Supplementary Table S1) (GTEx Consortium, 2013). IVs with low weak linkage disequilibrium (*r* ^2^ < 0.01) were selected within ±500 KB windows of the gene encodings for proxying antihypertensive drug targets, ensuring the robustness of selected IVs (Zheng et al., 2024). Moreover, the F-statistic approach was employed to mitigate the bias of IVs, using a criterion of an F-value>10 (Burgess and Thompson, 2011). Additionally, systolic blood pressure (N = 1,028,980) was used as a positive control to validate the relationship between IVs and antihypertensive drug targets (Supplementary Table S1) (Keaton et al., 2024).

### Outcome data

Two distinct CCRCC cohorts were utilized in this study to determine the association between antihypertensive drug targets and CCRCC risk. The discovery cohort was drawn from the United Kingdom Biobank and comprised 752,817 participants, whereas the validation cohort originated from Finland and included 315,137 participants (Supplementary Table S1) (Purdue et al., 2024; Kurki et al., 2023). CCRCC diagnosis was performed in accordance with the National Comprehensive Cancer Network (NCCN) guidelines (Stewart et al., 2014). Additionally, we performed an overlap check between the exposure and outcome databases, confirming the absence of no overlap among participants. This step effectively mitigated the risk of Type I errors and ensured the robustness of the MR analysis (Xie et al., 2022).

### Colocalization analysis

Colocalization analysis, a method based on Bayesian modeling to compute the posterior probability of association, was employed to assess potential confounding due to linkage disequilibrium between antihypertensive drug targets and risk of CCRCC arising from shared causal variants (Hukku et al., 2021). This method encompassed five key hypotheses: (1) neither the antihypertensive drug targets nor risk of CCRCC contained a causal variant within the genomic locus (H0); (2) only the antihypertensive drug targets carried a causal variant (H1); (3) only risk of CCRCC carried a causal variant (H2); (4) both the antihypertensive drug targets and CCRCC risk had distinct causal variants (H3); and (5) both the antihypertensive drug targets and CCRCC risk had a shared causal variant (H4) (Zuber et al., 2022). The prior probability that a SNP is associated with the antihypertensive drug target was set at 1 × 10^−4^ (p1), while the prior probability that a SNP was associated with the risk of CCRCC was also set at 1 × 10^−4^ (p2) (Sun et al., 2023). Additionally, the prior probability that a SNP is simultaneously associated with both the antihypertensive drug target andCCRCC risk was set at 1 × 10^−5^ (p12) (Sun et al., 2023). In the screening process, a 500 kb region upstream and downstream of each IV was selected for the drug targeted MR analysis (Liu et al., 2024). Furthermore, a posterior probability for shared causal variants (PP4) ≥ 0.8 was considered for two signals (Yang et al., 2024). Colocalization analysis was conducted using the “*coloc*” package (version 5.2.1), and the results were visualized using the “*LocusCompareR*” package (version 1.0.0).

### Sensitivity analysis

The heterogeneity in dependent instruments (HEIDI) test was utilized in this SMR study, leveraging multiple SNPs within a genomic locus to determine whether the relationship of antihypertensive drug targets and the risk of CCRCC were due to a shared genetic variant or genetic linkage (Wu et al., 2018). A HEIDI test with a P-value greater than 0.01 indicated that the association observed between antihypertensive drug targets and CCRCC risk was not confounded by linkage disequilibrium (Chauquet et al., 2021).

### Phenome-wide association analysis

A phenome-wide association study (PheWAS) was conducted utilizing data from the PheWeb database (https://pheweb.org/), encompassing approximately 15,500 binary and 1,500 continuous phenotypes, to systematically investigate the pleiotropic influences of candidate therapeutic targets and their potential adverse outcomes (Gagliano Taliun et al., 2020). Individuals within the exome sequencing subgroup were derived from the United Kingdom Biobank cohort. Detailed descriptions of the methodological framework underlying this analysis are available in the original publication (Wang et al., 2021).

### Western blotting

Cancerous and adjacent non-cancerous tissue samples were collected from three patients with CCRCC at the First Affiliated Hospital of Wenzhou Medical University. The tissues were lysed in a buffer consisting of RIPA buffer, protease inhibitor cocktail, and phenylmethylsulfonyl fluoride (PMSF) at a ratio of 100:10:1. Protein concentrations were measured using the bicinchoninic acid (BCA) assay. Equal amounts of protein were separated by 10% sodium dodecyl sulfate–polyacrylamide gel electrophoresis (SDS–PAGE) and transferred onto polyvinylidene difluoride (PVDF) membranes. Membranes were blocked with QuickBlock™ blocking buffer for 30 min and incubated overnight at 4 °C with primary antibodies against ADRB1 (1:1,000 dilution, catalog no. 28323-1-AP, Proteintech) and β-actin (1:1,000 dilution, catalog no. 66009-1-Ig, Proteintech). β-actin was used as a loading control. After incubation with primary antibodies, the membranes were incubated with horseradish peroxidase (HRP)-conjugated secondary antibodies for 1 h at room temperature. Signals were detected using SuperSignal™ West Femto Maximum Sensitivity Substrate (Thermo Scientific, United States). Bands were visualized and analyzed using a chemiluminescence imaging system.

### Prognostic analysis of tumor survival

A total of 532 patients with CCRCC, including complete RNA-seq profiles and clinical data were obtained from The Cancer Genome Atlas (TCGA; https://portal.gdc.cancer.gov/). Raw RNA-seq read counts were normalized to transcripts per kilobase million (TPM) and log2-transformed. Violin plots were constructed to display ADRB1 expression across different CCRCC stages. Box plots were constructed to illustrate median expression levels among tumor stages, and the Wilcoxon rank sum test was used to evaluate significance. The association between ADRB1 expression and tumor stage was further examined using scatter plots with boxplots, and hazard ratios for ADRB1 expression were estimated using density plots. Kaplan–Meier survival curves were generated to compare OS between patients with high and low ADRB1 expression, and survival differences were evaluated using the log-rank test.

### Statistical analysis

All analyses were performed using R software (version 4.3.1) and SMR software (version 1.3.1) (Zhu et al., 2016). To minimize bias, we applied a Bonferroni-corrected significance level, with a P-value threshold of <0.0125 (0.05/4, accounting for the four antihypertensive drug targets analyzed in relation to CCRCC risk) (Sedgwick, 2014). Associations with P-values between 0.0125 and 0.05 were considered suggestive, while P-values greater than 0.05 indicated no statistically significant association between antihypertensive drug targets and CCRCC risk.

## Results

### Positive control analysis

We initially calculated the F-statistics for each selected instrumental variable, retaining only those with F-values greater than 10 to minimize bias associated with weak instruments (Supplementary Table S2). Additionally, positive controls were employed to validate the reliability of the instrumental variables, with the results demonstrating that ACE, ADRB1, ADRB2, and SLC12A3 were significantly associated with systolic blood pressure (P < 0.05), confirming their credibility as targets for antihypertensive therapies (Supplementary Table S3).

### SMR analysis

**FIGURE 2:**
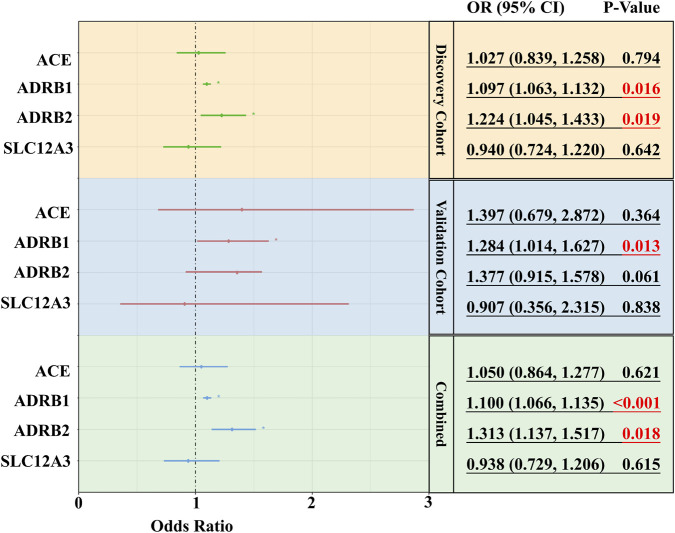
Results of summary-data-based Mendelian randomization analyses on antihypertensive drug targets and Clear Cell Renal Cell Carcinoma risk.

The results of the SMR analyses assessing the relationship of antihypertensive drug targets and CCRCC risk are presented in Table 1 and Figure 2. The analyses revealed a genetic link between ADRB1 and CCRCC risk in both the discovery cohort (OR = 1.097; 95%CI: 1.063–1.132; P = 0.016) and the validation cohort (OR = 1.284; 95%CI: 1.014–1.627; P = 0.013). Additionally, an association between ADRB2 and CCRCC risk was identified in the discovery cohort (OR = 1.224; 95%CI: 1.045–1.433; P = 0.019). In the combined cohort, ADRB1 (OR = 1.100; 95% CI: 1.066–1.135; P < 0.0001) and ADRB2 (OR = 1.313; 95%CI: 1.137–1.517; P = 0.0002) were significantly associated with CCRCC risk. However, no evidence supported an association between ACE or SLC12A3 and CCRCC risk (Table 1).

**TABLE 1:** Results of Summary-data-based Mendelian randomization analyses on antihypertensive drug targets and risks of Clear Cell Renal Cell Carcinoma.

Cancer	Gene	Discovery cohort		Validation cohort		Combined	
		Or (95% CI)	P-value	Or (95% CI)	P-value	Or (95% CI)	P-value
Clear cell renal cell carcinoma	ACE	1.027 (0.839, 1.258)	0.794	1.397 (0.679, 2.872)	0.364	1.050 (0.864, 1.277)	0.621
	ADRB1	1.097 (1.063, 1.132)	**0.016**	1.284 (1.014, 1.627)	**0.013**	1.100 (1.066, 1.135)	**<0.0001**
	ADRB2	1.224 (1.045, 1.433)	**0.019**	1.877 (1.315, 2.678)	0.061	1.313 (1.137, 1.517)	**0.0002**
	SLC12A3	0.940 (0.724, 1.220)	0.642	0.907 (0.356, 2.315)	0.838	0.938 (0.729, 1.206)	0.615

Bold values indicate statistically significant results (P < 0.05).

### Statistical power and sensitivity analyses

In both discovery and validation datasets, P-values from the HEIDI test of these SMR analyses were greater than 0.01, revealing no evidence of horizontal pleiotropy in this study (Supplementary Table S4). Furthermore, we assessed the power of the correlation analyses within the discovery and validation cohorts, and the results confirmed robust statistical performance (Supplementary Table S4).

### Colocalization analysis

**FIGURE 3:**
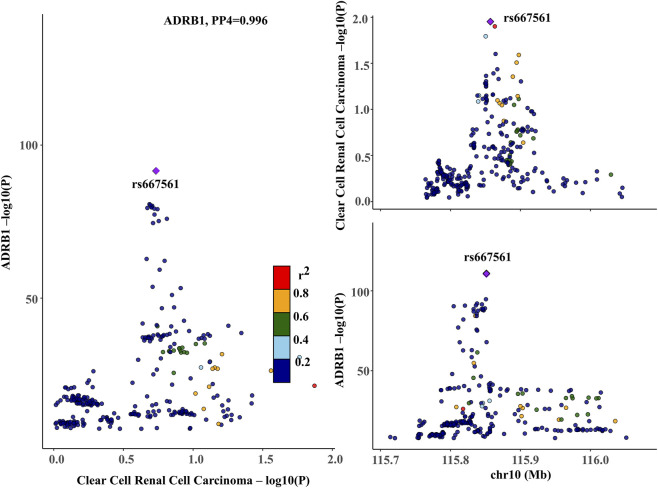
Regional Manhattan plot of associations of ADRB1 and risks of Clear Cell Renal Cell Carcinoma. The lead SNP is shown as a purple diamond. SNPs within ±500 kb of the antihypertensive drug target trait locus were included; p12 = 1 × 10^−5^, prior probability a SNP is associated with both ADRB1 and Clear Cell Renal Cell Carcinoma.

Colocalization analyses were performed to assess the likelihood of a shared causal variant between antihypertensive drug targets and CCRCC risk (Table 2). ADRB1 had shared genomic regions with CCRCC risk in two cohorts (PP4 = 0.996; 0.965) (Figure 3). Additionally, strong evidence supported the colocalization of ADRB2 and CCRCC risk (PP4 = 0.895) (Supplementary Figure S1). Conversely, no compelling evidence was found for colocalization between ACE, SLC12A3, and CCRCC risk in either the discovery or validation cohorts (Table 2; Supplementary Figures S2, S3).

**TABLE 2:** Colocalization analyses of antihypertensive drug targets and risk of Clear Cell Renal Cell Carcinoma in discovery and validation cohort.

Clear cell renal cell carcinoma	Gene	PP4
Discovery cohort	ACE	0.197
	ADRB1	0.996
	ADRB2	0.895
	SLC12A3	0.211
Validation cohort (Finland cohort)	ACE	0.201
	ADRB1	0.965
	ADRB2	0.712
	SLC12A3	0.218

### PheWAS analysis

PheWAS analysis revealed that, in addition to essential hypertension and hypertension, ADRB1 was significantly associated with several other conditions, including Eustachian tube disorders (P = 3.90 × 10^−8^), chronic venous insufficiency (P = 3.40 × 10^−7^), and hallux rigidus (P = 7.00 × 10^−7^) (Supplementary Table S5). These findings suggest that potential adverse effects involving these conditions should be considered when ADRB1 antagonists are used to treat CCRCC.

### Associations between ADRB1 and CCRCC

**FIGURE 4:**
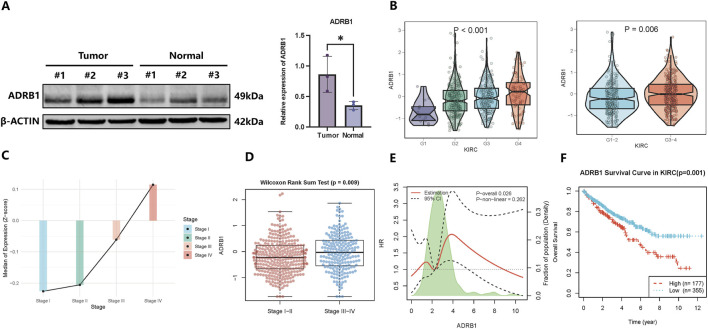
Associations between genetically predicted ADRB1 and Clear Cell Renal Cell Carcinoma (CCRCC). **(A)** ADRB1 protein expression in tumor and normal tissues (Mann-Whitney test, *P-value < 0.05); **(B)** ADRB1 expression across different stages and grades of CCRCC; **(C)** ADRB1 expression levels in different tumor stages; **(D)** Correlation between ADRB1 expression and tumor stages; **(E)** Hazard ratio estimation for ADRB1 expression in CCRCC; **(F)** ADRB1 survival curve in CCRCC.

Western blot quantification demonstrated a significant increase in ADRB1 levels in tumor tissues, suggesting a potential role in CCRCC pathogenesis (Figure 4A). Analysis across different tumor stages I-IV (P < 0.001) and grades (G1-2 vs. G3-4) revealed distinct expression patterns (P = 0.006) (Figure 4B). Figures 4C,D showed a positive correlation between ADRB1 expression and tumor stage (P = 0.009). The prognostic value of ADRB1 was evaluated by estimating hazard ratios using density plots, revealing a significant association with patient prognosis (P = 0.026) (Figure 4E). Kaplan-Meier survival analysis demonstrated that patients with high ADRB1 expression had significantly worse overall survival than those with low ADRB1 expression (P = 0.001) (Figure 4F).

## Discussion

This drug target MR study uncovered the genetic associations between four antihypertensive drug targets and the risk of CCRCC. The analyses revealed significant associations between ADRB1, ADRB2, and CCRCC risk in different cohorts. Additionally, colocalization analysis provided evidence that ADRB1 and ADRB2 share common causal variants with CCRCC in both cohorts. Furthermore, ADRB1 was highly expressed in CCRCC tumor tissues and was associated with poor survival and prognosis.

Beta-blockers are a first-line therapeutic option for cardiovascular diseases; however, their clinical benefits have shown variability across different studies, and for certain conditions, they are no longer considered the preferred treatment (Arnold, 2023). Some hypertension guidelines have recently downgraded the use of beta-blockers from their first-choice status (Mancia et al., 2022). Nonetheless, evidence supporting this reclassification may be insufficient, as beta-blockers, such as other major antihypertensive agents, effectively lower blood pressure and consistently reduce the risk of cardiovascular complications (Argulian et al., 2019). Beyond their cardiovascular effects, beta-blockers exert pharmacological effects by inhibiting beta-adrenergic receptors, which play a role in regulating the proliferation of cancer and immune cells (Blood Pressure Lowering Treatment Trialists Collaboration, 2014). This mechanism has led to increasing interest in beta-blockers as a potentially low-cost and relatively safe treatment option for certain cancers (Carnet Le Provost et al., 2023). For instance, a meta-analysis revealed that beta-blocker use is associated with improved recurrence-free survival (RFS) in patients with early-stage breast cancer, particularly in those with triple-negative breast cancer (Caparica et al., 2021). Numerous preclinical studies have also demonstrated that blocking adrenergic receptors can slow tumor growth, further reinforcing the potential clinical value of beta-blockers in cancer therapy (Tadic et al., 2019). These results suggest that beta-blockers may exhibit anticancer properties, either as standalone treatments or in combination with conventional anticancer therapies and immunotherapies. This finding is consistent with our own research, which highlights the need for future studies to focus on the antihypertensive drug targets ADRB1 and ADRB2 to further investigate the potential link between beta-blockers and the risk of CCRCC. While our results implicate both ADRB1 and ADRB2, the stronger colocalization evidence and consistent association across cohorts specifically highlight ADRB1. Unlike ADRB2, which is predominantly expressed in bronchial smooth muscle, ADRB1 is the primary β-adrenergic receptor in renal tissue, suggesting tissue-specific oncogenic mechanisms (Oliveira et al., 2026). Potential mechanisms include ADRB1-mediated activation of cAMP/PKA signaling promoting HIF-2α stabilization—a hallmark of CCRCC—or adrenergic stimulation of VEGF secretion enhancing tumor angiogenesis (Nowinka et al., 2026). Alternatively, ADRB1 signaling may modulate immune cell infiltration in the tumor microenvironment, given its role in regulating macrophage polarization (Sanganoo et al., 2026). Angiotensin-converting enzyme inhibitors (ACEIs), as cornerstones of first-line antihypertensive therapy, have attracted significant attention. A meta-analysis revealed that ACEI use may be associated with an elevated risk of lung cancer, with this risk being particularly pronounced in Asian populations (Wu et al., 2023). Similarly, another study posited a potential link between ACEIs and the development and progression of pancreatic cancer, although combination therapy with candesartan and gemcitabine did not markedly improve the prognosis of patients with pancreatic cancer (Asgharzadeh et al., 2022). These observations suggest a potential relationship between ACEIs and the pathogenesis of certain cancers. However, our study did not identify a significant correlation between ACEI targets and the risk of clear cell renal cell carcinoma (CCRCC), indicating that further in-depth investigations are warranted to elucidate the underlying mechanisms. Similarly, thiazide diuretics, another widely prescribed class of antihypertensive agents, have been linked to an increased risk of several malignancies, including skin cancer, gallbladder cancer, and colorectal cancer (Rouette et al., 2024; Stinton and Shaffer, 2012; Schneider et al., 2021; Birck et al., 2023). Nonetheless, a definitive association between thiazide diuretics and the risk of CCRCC, a finding that is consistent with our own results. These findings underscore the possibility that certain antihypertensive drug targets may contribute to the pathogenesis and progression of CCRCC has yet to be established, thereby offering new directions for future research aimed at unraveling the mechanistic pathways connecting antihypertensive medication use and CCRCC risk.

This drug target MR study has some limitations. Firstly, the study primarily focuses on individuals of European population, which may constrain the generalizability of the findings to other ethnic groups. Consequently, future research should broaden its scope to include a more diverse range of racial populations, thereby enhancing the robustness and applicability of the conclusions. Secondly, the development and progression of CCRCC is a prolonged and complex process. However, owing to the lack of detailed tumor staging data in the current CCRCC databases, we could not examine the association between antihypertensive drug targets and the distinct stages of CCRCC. Future research could delve into the different phases of CCRCC progression to better elucidate the interplay between disease evolution and therapeutic targets. Furthermore, we acknowledge that gene regulation is highly tissue-specific, and that ADRB1 expression in the renal cortex—the tissue relevant to CCRCC pathogenesis—may be governed by distinct cis-regulatory architectures that are not fully captured by blood eQTLs. However, previous work has demonstrated that cis-eQTLs for genes encoding drug targets frequently exhibit strong cross-tissue sharing, particularly when effect directions are concordant across blood and disease-relevant tissues. In our analysis, the exceptionally high colocalization posterior probability for ADRB1 suggests that the causal variant influencing ADRB1 expression in blood likely exerts a similar regulatory effect in renal tissue, thereby mitigating concerns about tissue mismatch. Nevertheless, validation using kidney cortex-specific eQTL data—such as from the GTEx kidney cortex or larger renal transcriptomic resources—would strengthen causal inference and should be pursued as these datasets expand.

Our study has several strengths. First, a drug target MR study based on SMR analyses was employed to elucidate the causal association between four antihypertensive drug targets and CCRCC risk. Second, we utilized the United Kingdom cohort as the discovery cohort and validated the findings in a Finnish cohort, with both populations consisting exclusively of individuals of European descent. This approach effectively minimizes bias arising from ethnic differences. Third, colocalization analyses were performed to determine the relationships between the drug targets and CCRCC risk. Finally, we discovered that high ADRB1 expression in tumor tissues was closely related to poor tumorigenesis and development. These results emphasized the significance of ADRB1 as a potential therapeutic target for CCRCC.

In conclusion, genetically predicted ADRB1 expression is associated with increased CCRCC risk and poor prognosis. These findings suggest that ADRB1 inhibition—already achieved with existing beta-blockers—may represent a repurposable therapeutic strategy for CCRCC prevention or treatment. Clinical trials investigating the use of beta-blockers in patients with high-risk renal mass, or the use of ADRB1 expression as a prognostic biomarker, are warranted.

## Data Availability

The original contributions presented in the study are included in the article/Supplementary Material, further inquiries can be directed to the corresponding author.
