# Supplementary material for: Genetic insights revealed ADRB1 as potential target for clear cell renal cell carcinoma
Source: Front Pharmacol. 2026 Jun 3;17:1838896. doi: 10.3389/fphar.2026.1838896 (PMC13272175; doi:10.3389/fphar.2026.1838896)
Supplement: Supplementary file 1 [file DataSheet1.pdf]

List of Supporting Information:

Supplementary Tables

Supplementary Table S1 | Detail of exposure and outcome data used in the study. **Abbreviations:** eQTL: expression quantitative trait loci; GWAS: genome-wide association study.

Supplementary Table S2 | Characteristics of SNPs extracted from exposure eQTL data associated with antihypertensive drug targets.

Supplementary Table S3 | SMR results of antihypertensive drug targets and systolic blood pressure in discovery cohort. **Abbreviations:** 95% CI: 95% confidence interval; eQTL: expression quantitative trait loci; GWAS: genome-wide association study; HEIDI: heterogeneity in dependent instruments; OR: odds ratio; SE: standard error; SMR: summary-data-based Mendelian rondomization; SNP: single nucleotide polymorphisms.

Supplementary Table S4 | Sensitivity and power analyses of Summary-data-based Mendelian randomization analyses in discovery and validation cohorts.

Supplementary Table S5 | Phenotypes with the most significant associations for ADRB1.

Supplementary Figure Legends

Supplementary Figure S1 | Regional Manhattan plot of associations of ADRB2 and risks of Clear Cell Renal Cell Carcinoma. The lead SNP is shown as a purple diamond. SNPs within ±500 kb of the antihypertensive drug target trait locus were included; $p12=1\times10-5$, prior probability a SNP is associated with both ADRB2 and Clear Cell Renal Cell Carcinoma.

Supplementary Figure S2 | Regional Manhattan plot of associations of ACE and risks of Clear Cell Renal Cell Carcinoma. The lead SNP is shown as a purple diamond. SNPs within ±500 kb of the antihypertensive drug target trait locus were included; $p12=1\times10-5$, prior probability a SNP is

associated with both ACE and Clear Cell Renal Cell Carcinoma.

Supplementary Figure S3 | Regional Manhattan plot of associations of SLC12A3 and risks of Clear Cell Renal Cell Carcinoma. The lead SNP is shown as a purple diamond. SNPs within ±500 kb of the antihypertensive drug target trait locus were included; $p12=1\times10-5$, prior probability a SNP is associated with both SLC12A3 and Clear Cell Renal Cell Carcinoma.

Supplementary Table S1 | Detail of exposure and outcome data used in the study.

Characteristic	Resource/Author	Sample Size	Population	PubMed ID	Download Source
			**Exposure**		
**eQTL data**					
eQTL for ADRB1, ADRB2, and SLC12A3	eQTLGen Consortium	31,684	Predominantly European	34475573	https://www.eqtlgen.org
eQTL for ACE	GTExV8	15,201	Predominantly European	29022597	https://www.gtexportal.org/home
			**Outcome**		
**Discovery Cohort**					
Clear Cell Renal Cell Carcinoma	Purdue MP et al.	752,817	European	38671320	https://www.ebi.ac.uk/gwas/
**Validation Cohort (Finland cohort)**					
Clear Cell Renal Cell Carcinoma	Mitja I Kurki et al.	315,137	European	36653562	https://www.finngen.fi/en
**GWAS summary data for positive control analysis**					
Systolic blood pressure	Keaton JM et al.	1,028,980	European	38689001	https://www.ebi.ac.uk/gwas/

Abbreviations: eQTL: expression quantitative trait loci; GWAS: genome-wide association study.

Supplementary Table S2 | Characteristics of SNPs extracted from exposure eQTL data associated with antihypertensive drug targets.

Gene	SNP	Gene base pair	Effect allele	Other allele	Beta	SE	F-statistic	P-Value
ADRB2	rs2082395	148200600	A	G	0.126274	0.0080146	248.23318	6.306E-56
ADRB2	rs11959615	148201316	T	A	0.12624	0.0080285	247.24611	1.036E-55
ADRB2	rs11168066	148201255	C	A	0.126227	0.0080277	247.24323	1.036E-55
ADRB2	rs34064454	148201569	A	G	0.126181	0.0080277	247.06183	1.135E-55
ADRB2	rs11958940	148201485	A	T	0.126028	0.0080277	246.46366	1.532E-55
ADRB2	rs9325120	148200958	C	A	0.125514	0.0080226	244.76645	3.594E-55
ADRB2	rs56330463	148200011	T	C	0.12455	0.0080386	240.06318	3.808E-54
ADRB2	rs11746634	148202668	C	G	0.12435	0.0080312	239.73757	4.491E-54
ADRB2	rs1432622	148203762	T	C	0.124085	0.0080182	239.48619	5.086E-54
ADRB2	rs11168067	148202801	A	G	0.124216	0.0080315	239.19794	5.885E-54
ADRB2	rs1432623	148204008	C	T	0.123985	0.0080181	239.10928	6.147E-54
ADRB2	rs11168068	148204121	C	T	0.12387	0.0080177	238.69212	7.591E-54
ADRB2	rs2400707	148205052	A	G	0.123758	0.0080231	237.93709	1.108E-53
ADRB2	rs1042711	148206348	C	T	0.123557	0.0080448	235.88769	3.102E-53
ADRB2	rs2053044	148205372	A	G	0.123087	0.0080233	235.35047	4.056E-53
ADRB2	rs1801704	148206375	C	T	0.123051	0.0080342	234.57911	5.978E-53
ADRB2	rs10078004	148199242	G	A	0.123553	0.0080721	234.28083	6.951E-53
ADRB2	rs11168070	148205927	G	C	0.123247	0.0080546	234.13705	7.46E-53
ADRB2	rs2082382	148200553	G	A	0.123264	0.0080737	233.09254	1.261E-52

ADRB2	rs11959427	148206028	C	T	0.122815	0.0080536	232.55168	1.656E-52
ADRB2	rs1042714	148206473	G	C	0.122851	0.0080879	230.71861	4.157E-52
ADRB2	rs4705059	148198999	C	T	0.120579	0.0081401	219.42441	1.207E-49
ADRB2	rs9325122	148202936	C	T	0.119975	0.0081375	217.36942	3.393E-49
ADRB2	rs12189018	148201005	C	T	-0.11664	0.0081787	203.39351	3.797E-46
ADRB2	rs17778257	148204577	T	A	-0.11528	0.0081658	199.28563	2.993E-45
ADRB2	rs12654778	148205741	A	G	-0.11514	0.0081715	198.55565	4.314E-45
ADRB2	rs35283004	148199267	G	A	-0.11607	0.0082517	197.868	6.093E-45
ADRB2	rs1042713	148206440	A	G	-0.1122	0.008204	187.02284	1.42E-42
ADRB2	rs67339154	148199445	A	G	0.115378	0.008655	177.71082	1.533E-40
ADRB2	rs4705060	148199004	G	A	0.111794	0.0087088	164.7845	1.02E-37
ADRB2	rs71582318	148199844	C	T	-0.10714	0.0090616	139.79759	2.948E-32
ADRB2	rs11960649	148203144	A	C	0.100915	0.0087137	134.12361	5.132E-31
ADRB2	rs2116715	148213506	A	G	0.087234	0.0081596	114.29723	1.122E-26
ADRB2	rs145180583	148199734	T	G	-0.11366	0.0118461	92.055392	8.424E-22
ADRB2	rs17108817	148215902	C	T	0.075575	0.0080454	88.239778	5.798E-21
ADRB2	rs11948371	148203108	T	A	0.090179	0.010132	79.217027	5.565E-19
ADRB2	rs11957757	148216187	A	G	0.06313	0.0080537	61.443739	4.556E-15
ADRB2	rs11957351	148203104	C	T	0.084728	0.0111997	57.232426	3.873E-14
ADRB2	rs67003124	148232300	G	A	-0.12421	0.0164819	56.791663	4.846E-14
ADRB2	rs72829140	148240696	C	G	-0.12019	0.0161549	55.35318	1.007E-13
ADRB2	rs72829143	148241484	C	T	-0.11958	0.0160838	55.279176	1.046E-13
ADRB2	rs6580586	148242723	C	A	-0.09736	0.0132356	54.108098	1.898E-13

ADRB2	rs6580587	148300556	T	C	-0.16534	0.0240131	47.405981	5.771E-12
ADRB2	rs72829142	148240905	G	A	-0.11134	0.0171677	42.063132	8.838E-11
ADRB2	rs72829126	148227089	T	A	-0.11452	0.017801	41.388669	1.248E-10
ADRB2	rs10066266	148216319	G	A	0.052491	0.008169	41.288384	1.314E-10
ADRB2	rs72829145	148244427	C	T	-0.11026	0.0171669	41.252685	1.338E-10
ADRB2	rs74609137	148195328	A	C	-0.30138	0.0472346	40.709331	1.766E-10
ADRB2	rs1042719	148207447	C	G	-0.05734	0.0090324	40.302199	2.176E-10
ADRB2	rs1042720	148207633	A	G	-0.05029	0.0088199	32.510496	1.186E-08
ADRB2	rs145670550	148235423	C	A	-0.28607	0.0507445	31.780269	1.726E-08
ADRB2	rs10045453	148176161	T	C	0.05472	0.0099299	30.366682	3.576E-08
ADRB2	rs8192451	148208014	T	C	-0.27596	0.0503807	30.001857	4.316E-08
ADRB1	rs4917675	115799477	C	T	0.249181	0.0100003	620.87445	4.82E-137
ADRB1	rs12412523	115786838	C	T	0.245468	0.0099488	608.76806	2.07E-134
ADRB1	rs7074495	115793787	C	A	0.247517	0.010053	606.20386	7.49E-134
ADRB1	rs7915120	115785245	G	A	0.244287	0.0099447	603.41915	3.02E-133
ADRB1	rs55720524	115798184	T	G	0.343626	0.0140909	594.69538	2.38E-131
ADRB1	rs72823017	115795225	A	G	0.343141	0.0140832	593.66648	4E-131
ADRB1	rs56012176	115793237	G	A	0.34241	0.0140688	592.35051	7.73E-131
ADRB1	rs4619105	115795236	A	G	0.342287	0.0140852	590.54742	1.9E-130
ADRB1	rs7079542	115794898	G	A	0.342337	0.0140955	589.85696	2.69E-130
ADRB1	rs17875416	115796337	G	A	0.341958	0.0140901	589.00284	4.13E-130
ADRB1	rs72823016	115788111	C	T	0.34113	0.0140689	587.92177	7.08E-130
ADRB1	rs72823015	115786611	T	G	0.341002	0.0140724	587.18846	1.02E-129

ADRB1	rs72823013	115786233	A	G	0.339398	0.0141029	579.16419	5.7E-128
ADRB1	rs72823014	115786236	A	G	0.33702	0.014083	572.69183	1.46E-126
ADRB1	rs72823020	115797947	A	T	0.286078	0.0141807	406.98048	1.664E-90
ADRB1	rs12355850	115826386	T	C	0.223212	0.0111769	398.83416	9.886E-89
ADRB1	rs4359161	115826508	A	G	0.220486	0.0111376	391.9032	3.185E-87
ADRB1	rs12777442	115780690	C	T	0.189688	0.0100667	355.06304	3.341E-79
ADRB1	rs4918889	115830718	G	C	0.212733	0.0115401	339.82129	6.985E-76
ADRB1	rs68122733	115831533	G	A	0.21266	0.0115872	336.83298	3.124E-75
ADRB1	rs686239	115844949	T	C	0.216285	0.0124051	303.98508	4.46E-68
ADRB1	rs72823019	115795716	A	C	0.421564	0.0289186	212.50664	3.9E-48
ADRB1	rs11196628	115850375	A	C	0.163543	0.0119797	186.3683	1.975E-42
ADRB1	rs540609	116040001	G	A	0.160375	0.0118243	183.9596	6.618E-42
ADRB1	rs17776203	115848372	C	A	0.117822	0.0087105	182.96441	1.092E-41
ADRB1	rs2530322	116028335	A	G	0.12681	0.0093921	182.29826	1.526E-41
ADRB1	rs677062	115853630	T	C	0.155052	0.0114922	182.03227	1.743E-41
ADRB1	rs35361716	115848797	G	A	0.122138	0.0092287	175.1542	5.539E-40
ADRB1	rs572208	116019598	G	A	0.15721	0.0119064	174.34095	8.342E-40
ADRB1	rs1801253	115805056	G	C	-0.13048	0.009894	173.92456	1.029E-39
ADRB1	rs2484294	115792062	G	A	-0.12847	0.009791	172.16001	2.496E-39
ADRB1	rs2773469	115798895	A	G	-0.1279	0.0097741	171.23303	3.98E-39
ADRB1	rs740746	115792787	G	A	-0.12776	0.0097718	170.9352	4.624E-39
ADRB1	rs7917869	115849223	C	T	0.116638	0.0089378	170.30318	6.355E-39
ADRB1	rs594751	115864780	T	G	0.148289	0.0113773	169.87891	7.859E-39
ADRB1	rs7076938	115789375	C	T	-0.12862	0.0098686	169.87004	7.901E-39

ADRB1	rs675298	115920298	T	C	0.14772	0.0113562	169.20474	1.105E-38
ADRB1	rs643148	115894242	C	T	0.14657	0.0112748	168.99447	1.227E-38
ADRB1	rs11196630	115850706	T	C	0.115507	0.008893	168.7023	1.42E-38
ADRB1	rs2782977	115875026	T	G	0.146727	0.0113043	168.47394	1.593E-38
ADRB1	rs72823046	115909057	C	T	0.149839	0.0115545	168.16957	1.858E-38
ADRB1	rs7905846	115850176	A	G	0.115242	0.0088935	167.91098	2.117E-38
ADRB1	rs12411734	115918789	A	G	0.148463	0.0114698	167.54242	2.547E-38
ADRB1	rs639870	116008299	T	C	0.144128	0.0111485	167.13363	3.129E-38
ADRB1	rs1329829	115965449	T	G	0.144036	0.011159	166.60635	4.078E-38
ADRB1	rs2782978	115780121	T	C	0.114315	0.008859	166.50949	4.284E-38
ADRB1	rs609333	115901572	C	G	0.145819	0.0113015	166.47769	4.352E-38
ADRB1	rs11196589	115779717	C	A	0.114161	0.0088538	166.25629	4.865E-38
ADRB1	rs2616657	115959622	G	A	0.143345	0.0111393	165.59579	6.779E-38
ADRB1	rs642602	115978429	T	C	0.143552	0.0111577	165.52711	7.011E-38
ADRB1	rs582413	115873670	C	T	0.145334	0.0113192	164.85533	9.838E-38
ADRB1	rs600295	115936797	G	A	0.144095	0.0112271	164.72619	1.049E-37
ADRB1	rs11196629	115850584	C	T	0.114354	0.0089138	164.57922	1.131E-37
ADRB1	rs601303	115936546	T	G	0.144374	0.0112551	164.54294	1.152E-37
ADRB1	rs17091414	115962953	G	A	0.142291	0.0111132	163.93686	1.561E-37
ADRB1	rs2447634	115960479	C	A	0.142556	0.0111426	163.68087	1.776E-37
ADRB1	rs7922287	115850305	A	T	0.114171	0.0089292	163.4875	1.958E-37
ADRB1	rs6585264	115849597	C	T	0.114034	0.0089189	163.47267	1.973E-37

ADRB1	rs11196647	115930251	A	T	0.145672	0.0114461	161.97094	4.197E-37
ADRB1	rs74236192	115996660	A	G	0.141771	0.0111613	161.34119	5.764E-37
ADRB1	rs646294	116028592	T	G	0.156302	0.0123206	160.9405	7.053E-37
ADRB1	rs639813	116008250	C	A	0.140123	0.0110898	159.65092	1.347E-36
ADRB1	rs660101	115896451	C	G	0.110616	0.0088387	156.62571	6.181E-36
ADRB1	rs476525	116034874	T	C	0.157608	0.0126238	155.87494	9.014E-36
ADRB1	rs606682	115889872	G	A	0.110218	0.0088349	155.63197	1.02E-35
ADRB1	rs72823039	115864299	A	G	0.28753	0.0233912	151.09891	9.975E-35
ADRB1	rs2616637	115968431	A	C	0.107807	0.0087957	150.22843	1.545E-34
ADRB1	rs79972777	115962455	G	A	0.285394	0.0233321	149.61737	2.102E-34
ADRB1	rs2447633	115966755	T	C	0.10746	0.0087949	149.29039	2.479E-34
ADRB1	rs998334	115967012	A	G	0.107379	0.008795	149.06372	2.776E-34
ADRB1	rs672785	116003053	C	T	0.106276	0.0087934	146.06789	1.255E-33
ADRB1	rs2616636	115972067	A	G	0.106493	0.008816	145.91566	1.354E-33
ADRB1	rs666429	115990783	G	A	0.106117	0.0088124	145.00616	2.142E-33
ADRB1	rs6585266	115929148	G	T	0.106685	0.008877	144.43724	2.851E-33
ADRB1	rs2927513	115958464	A	G	0.105298	0.0087854	143.65349	4.228E-33
ADRB1	rs584353	116005064	C	A	0.105499	0.0088104	143.38557	4.839E-33
ADRB1	rs1903890	115967869	T	G	0.10528	0.0088064	142.92096	6.113E-33
ADRB1	rs630051	116001796	C	A	0.10521	0.0088104	142.60107	7.183E-33
ADRB1	rs489262	116018945	T	A	0.105779	0.0089255	140.45453	2.116E-32
ADRB1	rs7901584	115849257	G	A	-0.10122	0.0086874	135.76579	2.247E-31

ADRB1	rs623499	115780369	T	G	0.10088	0.0087135	134.03644	5.365E-31
ADRB1	rs4917676	115822543	C	T	-0.13912	0.0121037	132.10268	1.421E-30
ADRB1	rs11196661	115987931	C	A	0.269873	0.0234962	131.92394	1.555E-30
ADRB1	rs10885533	115839616	G	A	-0.09876	0.0086827	129.38603	5.583E-30
ADRB1	rs7078339	115837395	A	G	-0.09854	0.0086837	128.76334	7.64E-30
ADRB1	rs7083211	115838431	A	T	-0.0957	0.0087139	120.62184	4.624E-28
ADRB1	rs526930	116025322	G	A	0.099403	0.009054	120.5384	4.822E-28
ADRB1	rs682836	115893434	G	A	-0.095	0.0086595	120.34517	5.315E-28
ADRB1	rs2781810	116022102	C	T	0.098931	0.0090739	118.87127	1.118E-27
ADRB1	rs634997	116021753	A	G	0.09889	0.0090743	118.76207	1.181E-27
ADRB1	rs2616656	115958567	G	C	-0.09348	0.0086825	115.91118	4.971E-27
ADRB1	rs143854972	115843445	A	G	0.208492	0.0194383	115.04354	7.702E-27
ADRB1	rs660601	115896583	A	G	-0.09369	0.0087368	115.0044	7.853E-27
ADRB1	rs597371	116032519	G	A	0.092968	0.0087585	112.66986	2.549E-26
ADRB1	rs667561	116016472	G	A	-0.09111	0.0086733	110.35933	8.175E-26
ADRB1	rs17875480	115810745	T	C	0.267244	0.0265328	101.44946	7.332E-24
ADRB1	rs79327327	115847055	G	A	0.183503	0.0185526	97.831064	4.555E-23
ADRB1	rs17661319	115825464	C	T	0.128936	0.0130426	97.728231	4.797E-23
ADRB1	rs649785	116022753	G	C	-0.08499	0.0087943	93.404511	4.263E-22
ADRB1	rs7902873	115894959	C	G	-0.08434	0.0087942	91.97969	8.757E-22
ADRB1	rs61335308	115824270	G	A	0.125251	0.0130985	91.436433	1.153E-21
ADRB1	rs12571953	115823916	T	A	0.125217	0.0131194	91.09586	1.369E-21

ADRB1	rs10885531	115814392	T	C	-0.08258	0.0086625	90.8741	1.531E-21
ADRB1	rs10787516	115813924	T	C	-0.08252	0.0086615	90.771354	1.613E-21
ADRB1	rs12569745	115823745	G	C	0.124627	0.013128	90.121237	2.241E-21
ADRB1	rs10787518	115821878	A	T	-0.08176	0.008696	88.389988	5.374E-21
ADRB1	rs117173140	115746343	G	A	-0.15847	0.0284967	30.924663	2.682E-08
ADRB1	rs2480554	115948463	G	A	-0.08029	0.0088706	81.916946	1.419E-19
ADRB1	rs4918887	115820177	A	G	0.111928	0.0125787	79.178263	5.676E-19
ADRB1	rs11196618	115820991	A	C	0.111918	0.0125787	79.164115	5.717E-19
ADRB1	rs78983771	115749276	G	A	0.819722	0.0922259	79.000052	6.211E-19
ADRB1	rs2050394	115812793	C	T	0.11138	0.0125867	78.305211	8.831E-19
ADRB1	rs12266538	115991745	A	G	0.130364	0.014805	77.535132	1.304E-18
ADRB1	rs11196611	115808988	A	G	0.110624	0.0125835	77.285106	1.48E-18
ADRB1	rs11196613	115809581	T	C	0.110074	0.0125383	77.071211	1.649E-18
ADRB1	rs7920400	115811141	G	A	0.109715	0.0125349	76.610848	2.081E-18
ADRB1	rs3813719	115806882	T	C	0.11008	0.0125857	76.500116	2.203E-18
ADRB1	rs7919873	115810668	C	A	0.109387	0.0125106	76.449587	2.258E-18
ADRB1	rs7907426	115808264	C	A	0.109371	0.0125164	76.356409	2.368E-18
ADRB1	rs2429511	115801253	C	T	-0.07554	0.0086586	76.118726	2.671E-18
ADRB1	rs3851555	115822101	G	T	0.109256	0.0125262	76.076743	2.729E-18
ADRB1	rs7085562	115755904	G	A	0.075626	0.0086899	75.738701	3.238E-18
ADRB1	rs61191616	115821711	A	C	0.108699	0.0126031	74.386878	6.421E-18
ADRB1	rs17577566	115819073	C	G	0.108292	0.0126068	73.787539	8.697E-18

ADRB1	rs2782982	115788495	G	A	-0.07454	0.00868	73.736478	8.927E-18
ADRB1	rs657497	115899972	A	G	0.091507	0.0106663	73.599743	9.57E-18
ADRB1	rs11196617	115818295	A	G	0.108065	0.0126061	73.486679	1.014E-17
ADRB1	rs2050393	115814952	A	G	0.10781	0.0126055	73.147239	1.203E-17
ADRB1	rs4917671	115794895	A	G	0.109408	0.0127978	73.084877	1.242E-17
ADRB1	rs12359003	115814001	A	G	0.107848	0.0126179	73.055014	1.261E-17
ADRB1	rs2183378	115808892	T	C	0.107682	0.0126165	72.846458	1.402E-17
ADRB1	rs7476308	115813347	T	G	0.106992	0.012543	72.761186	1.464E-17
ADRB1	rs11196612	115809014	A	G	0.107471	0.0125997	72.754887	1.469E-17
ADRB1	rs3863275	115815033	A	G	0.107447	0.012604	72.672784	1.531E-17
ADRB1	rs2050395	115801595	G	T	0.106686	0.0125186	72.627875	1.566E-17
ADRB1	rs10787515	115790005	C	T	-0.07381	0.0086798	72.307701	1.841E-17
ADRB1	rs2024183	115817760	T	C	0.107045	0.012595	72.233129	1.912E-17
ADRB1	rs11196615	115810411	G	C	0.106763	0.0125686	72.155215	1.99E-17
ADRB1	rs7917421	115797866	G	A	0.106804	0.0126008	71.842064	2.331E-17
ADRB1	rs17875439	115801750	C	T	0.106678	0.0125943	71.746656	2.446E-17
ADRB1	rs11196614	115809978	T	C	0.106511	0.0125753	71.738487	2.458E-17
ADRB1	rs730473	115754129	T	G	0.074055	0.0087457	71.700195	2.505E-17
ADRB1	rs7922061	115822025	G	A	0.106316	0.0125574	71.679968	2.531E-17
ADRB1	rs17875432	115800251	T	C	0.10639	0.0125965	71.334864	3.015E-17
ADRB1	rs11196601	115795987	C	T	0.106346	0.0125973	71.266819	3.12E-17
ADRB1	rs3851554	115821938	C	T	0.106028	0.0125602	71.260363	3.13E-17
ADRB1	rs11196596	115787415	A	G	0.104934	0.0124607	70.916546	3.727E-17
ADRB1	rs10885530	115787889	T	C	0.104845	0.0124513	70.903236	3.752E-17

ADRB1	rs12569709	115800637	C	A	0.106216	0.012651	70.490432	4.627E-17
ADRB1	rs35818742	115793989	C	T	0.10513	0.0125355	70.334759	5.004E-17
ADRB1	rs9664370	115788553	G	A	0.104174	0.0124512	69.999713	5.93E-17
ADRB1	rs7090339	115784493	G	A	0.104051	0.0124377	69.986192	5.971E-17
ADRB1	rs80128970	115851893	A	G	0.145712	0.0174522	69.709228	6.873E-17
ADRB1	rs7917912	115798187	T	C	0.104507	0.0126225	68.548823	1.238E-16
ADRB1	rs855715	115823524	T	G	-0.12265	0.0148461	68.251208	1.44E-16
ADRB1	rs9663133	115788312	A	C	0.10264	0.0124872	67.562102	2.042E-16
ADRB1	rs7096104	115786214	T	A	0.102383	0.0124657	67.456276	2.154E-16
ADRB1	rs2900998	115790823	C	G	0.103046	0.0125492	67.426434	2.186E-16
ADRB1	rs4918886	115794926	C	G	0.103454	0.0126164	67.239376	2.404E-16
ADRB1	rs17577066	115795689	A	G	0.103513	0.0126266	67.207377	2.444E-16
ADRB1	rs2150043	115751682	T	G	0.071554	0.0087306	67.171127	2.489E-16
ADRB1	rs11196602	115796104	C	T	0.103481	0.0126265	67.166894	2.493E-16
ADRB1	rs4917674	115799335	T	C	0.103492	0.012637	67.06958	2.621E-16
ADRB1	rs11196598	115793754	T	A	0.103397	0.0126267	67.05577	2.638E-16
ADRB1	rs12416480	115786830	A	C	0.102234	0.0124989	66.903235	2.853E-16
ADRB1	rs7093444	115794324	C	T	0.102206	0.0125103	66.744784	3.089E-16
ADRB1	rs34346890	115790909	C	G	0.101909	0.0124793	66.68753	3.183E-16
ADRB1	rs2900997	115790744	G	A	0.101901	0.0124885	66.578857	3.361E-16
ADRB1	rs12767463	115784740	C	G	0.101917	0.0124914	66.568847	3.378E-16
ADRB1	rs7895363	115793001	G	A	0.101891	0.0124885	66.56579	3.384E-16
ADRB1	rs17875414	115796312	T	C	0.103704	0.0127169	66.50105	3.498E-16

ADRB1	rs10885529	115785750	T	C	0.101885	0.0124961	66.477016	3.538E-16
ADRB1	rs12357112	115789709	C	T	0.101725	0.0124806	66.433092	3.621E-16
ADRB1	rs7090717	115784692	T	C	0.101588	0.0124672	66.396772	3.688E-16
ADRB1	rs11196595	115784376	C	T	0.101768	0.0124945	66.341413	3.793E-16
ADRB1	rs12414581	115789150	G	A	0.101621	0.0124884	66.214534	4.045E-16
ADRB1	rs117126153	115972052	C	T	0.357257	0.0439204	66.165092	4.147E-16
ADRB1	rs11196599	115793783	G	C	0.103293	0.0127318	65.82065	4.938E-16
ADRB1	rs11196600	115794127	T	C	0.101875	0.0125893	65.483529	5.86E-16
ADRB1	rs12355435	115789537	T	C	0.100869	0.01249	65.221466	6.693E-16
ADRB1	rs4917673	115796949	A	G	0.105145	0.0130349	65.067159	7.24E-16
ADRB1	rs4918895	116043915	G	C	0.05343	0.009552	31.288362	2.224E-08
ADRB1	rs11196604	115797036	T	C	0.101817	0.0126474	64.809413	8.252E-16
ADRB1	rs1801252	115804036	G	A	0.102013	0.0126923	64.599682	9.176E-16
ADRB1	rs6585257	115748612	A	G	0.069626	0.0086838	64.288347	1.075E-15
ADRB1	rs2782979	115781367	T	G	0.069601	0.0086823	64.262651	1.089E-15
ADRB1	rs117624845	115853789	A	G	0.133732	0.016694	64.172633	1.14E-15
ADRB1	rs10787521	116044587	T	C	0.052851	0.009511	30.877958	2.748E-08
ADRB1	rs10885532	115828256	C	G	-0.06929	0.0087295	63.008618	2.058E-15
ADRB1	rs10509997	115924321	C	T	0.12676	0.0165457	58.694062	1.842E-14
ADRB1	rs78833131	115849782	C	T	0.07786	0.0101951	58.323501	2.223E-14
ADRB1	rs55662965	116003544	A	G	0.123215	0.016316	57.029527	4.293E-14
ADRB1	rs139275657	115713932	G	A	-0.1714	0.0314261	29.746134	4.925E-08
ADRB1	rs1335072	115836142	A	T	-0.06532	0.0087197	56.114999	6.835E-14

ADRB1	rs10160173	115975065	G	A	0.122046	0.0163307	55.851842	7.815E-14
ADRB1	rs12572086	115819224	G	A	0.094766	0.0126869	55.794726	8.044E-14
ADRB1	rs7902330	115915526	A	T	0.123807	0.0165939	55.666442	8.587E-14
ADRB1	rs7080644	115849796	T	C	0.073952	0.0099302	55.460736	9.534E-14
ADRB1	rs75362186	115918168	C	T	0.123561	0.0165961	55.430748	9.68E-14
ADRB1	rs7075063	116007131	T	C	0.121137	0.0162736	55.409771	9.783E-14
ADRB1	rs76235992	115914293	A	G	0.122896	0.0165963	54.834381	1.312E-13
ADRB1	rs3888629	115982753	A	C	0.121427	0.0164002	54.819143	1.321E-13
ADRB1	rs12255410	116006120	T	C	0.120938	0.0163527	54.694874	1.408E-13
ADRB1	rs75971578	115763271	A	G	-0.14953	0.0255169	34.341881	4.623E-09
ADRB1	rs12262953	116014827	T	G	0.1201	0.0162707	54.484571	1.566E-13
ADRB1	rs56041166	116003474	T	C	0.120841	0.0163795	54.428622	1.612E-13
ADRB1	rs9665610	116008464	A	T	0.121084	0.0164203	54.376512	1.656E-13
ADRB1	rs11196656	115976111	G	A	0.120068	0.0163678	53.811356	2.207E-13
ADRB1	rs10159905	115977065	C	G	0.119688	0.0163661	53.482392	2.609E-13
ADRB1	rs34322536	115993814	A	G	0.119096	0.0163495	53.062218	3.231E-13
ADRB1	rs12256668	115931101	A	G	0.120731	0.0165757	53.051032	3.25E-13
ADRB1	rs3851557	116000669	C	G	0.119133	0.0163608	53.021875	3.299E-13
ADRB1	rs58093408	115973468	T	C	0.119188	0.016372	52.998258	3.338E-13
ADRB1	rs10160168	115974828	G	A	0.119048	0.0163721	52.87318	3.558E-13
ADRB1	rs12253699	115975457	A	G	0.118915	0.0163754	52.733846	3.82E-13
ADRB1	rs12268539	115962623	T	C	0.118616	0.0163451	52.663702	3.958E-13
ADRB1	rs78356924	116010832	T	C	0.15055	0.0207701	52.539308	4.217E-13

ADRB1	rs11196649	115942689	T	C	0.120311	0.0166443	52.249195	4.887E-13
ADRB1	rs1034257	115832597	A	G	-0.07311	0.0101401	51.990185	5.579E-13
ADRB1	rs2012317	115832692	G	C	-0.07316	0.0101563	51.891963	5.864E-13
ADRB1	rs3887692	115982626	A	G	0.118285	0.0164322	51.816482	6.092E-13
ADRB1	rs17578010	115988417	G	A	0.118159	0.0164266	51.741409	6.329E-13
ADRB1	rs1964173	115832782	A	G	-0.07293	0.0101669	51.460715	7.305E-13
ADRB1	rs11196621	115831756	G	C	-0.07276	0.0101567	51.317114	7.859E-13
ADRB1	rs1034258	115832408	G	A	-0.07256	0.0101373	51.231183	8.21E-13
ADRB1	rs1034259	115832294	T	C	-0.07258	0.010143	51.208325	8.307E-13
ADRB1	rs17091284	115831804	T	G	-0.0726	0.0101563	51.097096	8.792E-13
ADRB1	rs2419885	115832275	C	T	-0.07243	0.0101519	50.908638	9.679E-13
ADRB1	rs74613308	115964736	G	A	0.116983	0.016397	50.899875	9.721E-13
ADRB1	rs12268028	115947264	A	G	0.116948	0.0164083	50.799381	1.023E-12
ADRB1	rs78130910	116021805	T	C	0.116858	0.0164478	50.477898	1.205E-12
ADRB1	rs11196674	116010629	A	G	0.116653	0.0164249	50.44131	1.228E-12
ADRB1	rs11196673	116010525	A	G	0.116436	0.0164481	50.112155	1.452E-12
ADRB1	rs7080509	115849765	G	A	0.071201	0.0100586	50.106429	1.456E-12
ADRB1	rs55683285	115951785	C	T	0.11615	0.0164179	50.0499	1.499E-12
ADRB1	rs77625645	116022007	G	A	0.11634	0.0164648	49.928118	1.595E-12
ADRB1	rs2012324	115832822	T	G	-0.06219	0.0088777	49.069901	2.47E-12
ADRB1	rs7080511	115849731	A	C	0.07012	0.0100237	48.935215	2.645E-12
ADRB1	rs74236195	116027555	G	C	0.11516	0.0164675	48.904404	2.687E-12
ADRB1	rs76468627	116033892	T	C	0.114055	0.0164829	47.880801	4.529E-12

ADRB1	rs7098512	115849742	C	T	0.06811	0.0099092	47.243695	6.269E-12
ADRB1	rs12414657	115803375	C	T	0.08934	0.0129983	47.240756	6.278E-12
ADRB1	rs7098598	115828842	T	C	-0.0712	0.0103738	47.109119	6.715E-12
ADRB1	rs740744	115827840	C	G	-0.07075	0.0103293	46.917453	7.406E-12
ADRB1	rs6585265	115849725	T	C	0.067664	0.0098996	46.717185	8.2E-12
ADRB1	rs10490905	115782271	G	A	0.096157	0.0140729	46.687242	8.327E-12
ADRB1	rs12256663	115836046	A	G	-0.06942	0.0102463	45.905806	1.241E-11
ADRB1	rs12771397	115781902	T	G	0.095698	0.0141318	45.857445	1.272E-11
ADRB1	rs17875422	115798092	G	A	-0.13146	0.0240362	29.912246	4.521E-08
ADRB1	rs67234920	115782061	A	G	0.093221	0.0141047	43.681259	3.864E-11
ADRB1	rs77334369	115782368	A	G	0.093977	0.0142757	43.336227	4.611E-11
ADRB1	rs77768243	115782367	C	T	0.093872	0.0142806	43.209775	4.918E-11
ADRB1	rs74236194	116013101	T	C	0.10733	0.0163324	43.185927	4.978E-11
ADRB1	rs67728379	115782424	G	C	0.092654	0.0141095	43.122616	5.141E-11
ADRB1	rs117648943	115846302	G	A	0.513463	0.0783506	42.947081	5.624E-11
ADRB1	rs7069673	115783466	C	T	0.092337	0.0141071	42.84225	5.934E-11
ADRB1	rs659280	115899581	G	A	0.076827	0.0117642	42.648754	6.551E-11
ADRB1	rs7070157	115783832	C	T	0.091897	0.0141072	42.434689	7.308E-11
ADRB1	rs77030796	115748636	T	G	-0.12843	0.0200032	41.223754	1.357E-10
ADRB1	rs238746	115766996	A	C	-0.05641	0.0088073	41.026665	1.502E-10
ADRB1	rs2480792	115773296	A	G	-0.0562	0.0087834	40.934309	1.574E-10
ADRB1	rs11196627	115845762	C	T	-0.05713	0.0089372	40.860173	1.635E-10

ADRB1	rs17091184	115748088	T	G	-0.12742	0.0199968	40.600084	1.868E-10
ADRB1	rs4918881	115751002	T	C	-0.12929	0.0203677	40.297005	2.181E-10
ADRB1	rs10885527	115772285	T	C	-0.05664	0.0089447	40.091642	2.423E-10
ADRB1	rs17875473	115800294	T	C	-0.09259	0.0149151	38.534485	5.38E-10
ADRB1	rs7921036	115774142	T	C	-0.05534	0.0087779	39.747987	2.889E-10
ADRB1	rs758587	115767296	A	T	-0.05602	0.0089082	39.551527	3.195E-10
ADRB1	rs2532827	115774519	C	G	-0.05514	0.0088043	39.217621	3.791E-10
ADRB1	rs7087846	115769919	C	T	-0.05546	0.0088807	39.002473	4.233E-10
ADRB1	rs4918885	115778581	T	C	-0.05558	0.0089156	38.857757	4.558E-10
ADRB1	rs2480790	115768772	A	C	-0.05544	0.0089062	38.750666	4.816E-10
ADRB1	rs10749151	115777224	T	C	-0.05521	0.008887	38.591414	5.225E-10
ADRB1	rs390261	115765102	G	A	-0.05476	0.0088212	38.539338	5.366E-10
ADRB1	rs4918894	116040773	C	T	0.057413	0.0092501	38.524403	5.407E-10
ADRB1	rs10787512	115779327	T	C	-0.05498	0.0088862	38.286409	6.109E-10
ADRB1	rs1576477	115775856	A	G	-0.05497	0.0088863	38.259231	6.195E-10
ADRB1	rs10749156	115779317	G	A	-0.05512	0.0089139	38.241928	6.25E-10
ADRB1	rs10749155	115779283	A	C	-0.05508	0.0089144	38.182547	6.443E-10
ADRB1	rs11196587	115772799	T	C	-0.05507	0.0089162	38.152954	6.541E-10
ADRB1	rs2900995	115777783	A	G	-0.05486	0.0088865	38.113321	6.675E-10
ADRB1	rs2419884	115778137	A	C	-0.05499	0.0089143	38.049182	6.898E-10
ADRB1	rs758586	115767540	C	T	-0.05479	0.0088865	38.019592	7.004E-10
ADRB1	rs17095610	115747085	A	G	-0.1346	0.021836	37.994837	7.093E-10
ADRB1	rs4256930	115777836	A	G	-0.05484	0.0089144	37.847151	7.651E-10

ADRB1	rs2900996	115778161	A	C	-0.05481	0.0089147	37.802776	7.827E-10
ADRB1	rs9421158	115775432	C	T	-0.05476	0.0089105	37.768326	7.966E-10
ADRB1	rs10749154	115779106	T	C	-0.05472	0.0089141	37.677513	8.346E-10
ADRB1	rs10749150	115775443	A	T	-0.05471	0.0089141	37.675034	8.357E-10
ADRB1	rs10749153	115778982	T	C	-0.05471	0.0089141	37.66531	8.399E-10
ADRB1	rs10749152	115778928	G	A	-0.05468	0.0089142	37.621082	8.591E-10
ADRB1	rs2532826	115774712	G	A	-0.05465	0.0089121	37.603874	8.667E-10
ADRB1	rs1576478	115775553	T	C	-0.05461	0.0089143	37.535296	8.978E-10
ADRB1	rs11196584	115765270	C	T	-0.0538	0.0088172	37.236766	1.046E-09
ADRB1	rs10749157	115780129	C	T	-0.05434	0.0089138	37.163644	1.086E-09
ADRB1	rs10787513	115779796	G	A	-0.05419	0.008914	36.95909	1.206E-09
ADRB1	rs2532825	115780194	A	G	-0.05433	0.0089407	36.925101	1.228E-09
ADRB1	rs7350419	115765396	A	G	-0.0542	0.0089367	36.789024	1.316E-09
ADRB1	rs10787514	115779852	T	C	-0.05385	0.0088858	36.728586	1.358E-09
ADRB1	rs678669	115764032	C	T	-0.05319	0.0088007	36.522643	1.509E-09
ADRB1	rs77083475	115765269	A	G	-0.05276	0.0087949	35.980764	1.993E-09
ADRB1	rs10885545	116076578	A	G	0.070709	0.011848	35.61696	2.402E-09
ADRB1	rs4918884	115778547	A	G	-0.05324	0.0089304	35.545466	2.492E-09
ADRB1	rs1335071	115840117	G	C	-0.05484	0.0091994	35.540723	2.498E-09
ADRB1	rs7911312	115771504	T	C	-0.05287	0.0088842	35.409562	2.672E-09
ADRB1	rs7076871	115842297	T	C	-0.05346	0.0092003	33.765393	6.218E-09
ADRB1	rs2419883	115778094	G	C	-0.05158	0.0089992	32.855806	9.925E-09

ADRB1	rs717368	115707296	G	A	-0.08795	0.0153481	32.83976	1.001E-08
ADRB1	rs2782981	115781547	T	C	-0.05491	0.0096083	32.658958	1.098E-08
ADRB1	rs10787511	115747997	A	G	-0.04833	0.0086938	30.900206	2.716E-08
ADRB1	rs10787510	115747742	A	G	-0.04758	0.008686	30.01072	4.297E-08
ADRB1	rs79352661	115942905	C	T	-0.12088	0.022118	29.866289	4.629E-08
SLC12A3	rs12924698	56791999	G	T	0.077775	0.0118253	43.257154	4.8E-11
SLC12A3	rs8044753	56883438	G	A	0.075539	0.0118374	40.722498	1.755E-10
SLC12A3	rs1561140	56864398	C	T	0.076104	0.0118366	41.339427	1.279E-10
SLC12A3	rs28514610	56827454	T	C	0.076442	0.0118342	41.723487	1.051E-10
SLC12A3	rs10852551	56765103	T	C	0.074814	0.0118357	39.955084	2.599E-10
SLC12A3	rs13335179	56842961	A	C	0.076011	0.0118349	41.249434	1.34E-10
SLC12A3	rs4784727	56787107	T	C	0.075959	0.011839	41.164922	1.399E-10
SLC12A3	rs12599065	56896036	C	T	-0.07358	0.011991	37.657992	8.431E-10
SLC12A3	rs3829502	56896730	A	G	-0.0729	0.0120043	36.874131	1.26E-09
SLC12A3	rs1436424	56895034	T	G	-0.0742	0.0119583	38.496783	5.484E-10
SLC12A3	rs9938953	56826766	C	T	-0.07478	0.0120402	38.571484	5.278E-10
SLC12A3	rs9939678	56850602	G	A	-0.07544	0.0120408	39.250108	3.728E-10
SLC12A3	rs2007432	56874857	T	C	-0.07515	0.0120437	38.932846	4.387E-10
SLC12A3	rs7187512	56840171	G	A	-0.07482	0.0120431	38.594167	5.218E-10
SLC12A3	rs9929577	56836108	C	T	-0.07453	0.0121138	37.852185	7.632E-10
SLC12A3	rs7199480	56859595	A	G	-0.07522	0.0120414	39.020209	4.195E-10
SLC12A3	rs1529929	56849496	A	G	-0.07436	0.0120354	38.172611	6.475E-10
SLC12A3	rs4783959	56834096	G	A	-0.07475	0.0120445	38.519314	5.421E-10
SLC12A3	rs735144	56883924	G	A	-0.07518	0.0120365	39.017623	4.2E-10

SLC12A3	rs1865830	56874197	A	G	-0.07602	0.012061	39.722496	2.927E-10
SLC12A3	rs8045306	56883709	C	G	-0.07386	0.0120422	37.620807	8.591E-10
SLC12A3	rs2099536	56861995	G	C	-0.07526	0.0120267	39.162368	3.899E-10
SLC12A3	rs2399562	56880158	G	A	-0.07539	0.0120475	39.160014	3.905E-10
SLC12A3	rs8052978	56814847	A	G	-0.07422	0.0121018	37.61375	8.624E-10
SLC12A3	rs1561139	56852822	T	G	-0.07249	0.0120606	36.122287	1.853E-09
SLC12A3	rs4784730	56865249	A	C	-0.07116	0.0119616	35.386067	2.705E-09
SLC12A3	rs1347591	56868700	G	A	-0.07166	0.0119568	35.915946	2.06E-09
SLC12A3	rs4461062	56864618	T	C	-0.07142	0.0119555	35.681524	2.324E-09
SLC12A3	rs13338063	56838978	G	T	-0.07113	0.0120056	35.103473	3.127E-09
SLC12A3	rs6499855	56885138	G	T	-0.06899	0.0121762	32.105744	1.46E-08
SLC12A3	rs955513	56946072	C	T	-0.07639	0.0118684	41.432593	1.22E-10
SLC12A3	rs5805	56947522	A	G	-0.07348	0.0118985	38.135776	6.6E-10
SLC12A3	rs37030	56948248	C	T	-0.07586	0.0118933	40.684175	1.79E-10
SLC12A3	rs37029	56949168	A	G	-0.07612	0.0118941	40.954576	1.558E-10
SLC12A3	rs3764263	56965707	A	G	-0.07386	0.0118944	38.556662	5.319E-10
SLC12A3	rs37026	56950134	T	C	-0.07605	0.0118967	40.867974	1.629E-10
SLC12A3	rs9926292	56952588	T	C	-0.07629	0.0118962	41.121792	1.431E-10
SLC12A3	rs4548848	56957798	A	G	-0.07549	0.0118946	40.276591	2.204E-10
SLC12A3	rs9924286	56952811	A	C	-0.07631	0.0118962	41.147131	1.412E-10
SLC12A3	rs37031	56947721	C	T	-0.07451	0.0118889	39.272931	3.685E-10
SLC12A3	rs28168	56951276	A	G	-0.07641	0.0118926	41.278405	1.32E-10
SLC12A3	rs711746	56946804	T	C	-0.07466	0.0118959	39.390847	3.47E-10
SLC12A3	rs1428847	56969854	A	G	-0.07284	0.0118992	37.471679	9.276E-10
SLC12A3	rs37024	56966071	T	G	-0.07409	0.0119418	38.494471	5.491E-10

SLC12A3	rs39718	56969683	C	T	-0.07338	0.0119082	37.970525	7.183E-10
SLC12A3	rs2518056	56963647	A	G	-0.07544	0.0118997	40.193045	2.301E-10
SLC12A3	rs711749	56946421	T	C	-0.07575	0.0118909	40.584793	1.883E-10
SLC12A3	rs5808	56947981	G	C	-0.07237	0.0118912	37.034541	1.161E-09
SLC12A3	rs37027	56950028	G	C	-0.07501	0.0118966	39.75317	2.882E-10
SLC12A3	rs711747	56946647	C	G	-0.0744	0.0118866	39.17505	3.875E-10
SLC12A3	rs708270	56946384	C	T	-0.07527	0.0118945	40.040822	2.487E-10
SLC12A3	rs12596509	56946445	T	C	-0.07525	0.011895	40.020542	2.513E-10
SLC12A3	rs4567697	56945619	T	G	-0.07306	0.0118964	37.719116	8.169E-10
SLC12A3	rs12924331	56953261	G	A	0.067035	0.0119134	31.661144	1.836E-08
SLC12A3	rs3794648	56946546	G	C	-0.07412	0.0118918	38.845269	4.588E-10
SLC12A3	rs9921780	56952098	G	A	0.066556	0.0119152	31.200951	2.326E-08
SLC12A3	rs3812964	56948841	C	T	-0.07504	0.0118958	39.79563	2.819E-10
SLC12A3	rs9925265	56950210	A	G	0.066724	0.0119052	31.411462	2.087E-08
SLC12A3	rs3794647	56946581	G	A	-0.07529	0.0118871	40.119778	2.389E-10
SLC12A3	rs6499860	56944955	A	C	-0.07003	0.0119128	34.553116	4.147E-09
SLC12A3	rs711748	56946594	C	G	-0.07412	0.0118919	38.845349	4.588E-10
SLC12A3	rs11508026	56999328	T	C	-0.07039	0.0119605	34.640269	3.966E-09
SLC12A3	rs4784741	57001216	T	C	-0.06864	0.0119554	32.958936	9.412E-09
SLC12A3	rs12720926	56998918	G	A	-0.07062	0.0119596	34.866519	3.531E-09
SLC12A3	rs12444012	57001438	A	G	-0.06946	0.0119543	33.758449	6.24E-09
SLC12A3	rs193695	56985156	A	G	0.076263	0.0123679	38.022277	6.995E-10

SLC12A3	rs708272	56996288	A	G	-0.06904	0.0119301	33.487149	7.174E-09
SLC12A3	rs711752	56996211	A	G	-0.0697	0.0119302	34.131091	5.152E-09
SLC12A3	rs9989419	56985139	A	G	0.069751	0.0121121	33.163814	8.471E-09
SLC12A3	rs9938160	56984590	C	T	-0.08659	0.0129298	44.849943	2.127E-11
SLC12A3	rs1800775	56995236	A	C	-0.0847	0.0147296	33.06695	8.903E-09
SLC12A3	rs173538	56982299	T	C	-0.07477	0.0125292	35.610103	2.411E-09
SLC12A3	rs37025	56966011	G	A	-0.07184	0.0125692	32.668157	1.093E-08
SLC12A3	rs3764261	56993324	A	C	-0.10259	0.012695	65.302185	6.424E-16
SLC12A3	rs247616	56989590	T	C	-0.10377	0.012736	66.382319	3.712E-16
SLC12A3	rs183130	56991363	T	C	-0.10331	0.0127049	66.116307	4.251E-16
SLC12A3	rs247617	56990716	A	C	-0.10379	0.0127001	66.789026	3.023E-16
SLC12A3	rs17231506	56994528	T	C	-0.10322	0.0127182	65.869569	4.818E-16
SLC12A3	rs173539	56988044	T	C	-0.10399	0.0127992	66.01499	4.474E-16
SLC12A3	rs56156922	56987369	C	T	-0.10641	0.0128668	68.389769	1.342E-16
SLC12A3	rs12446515	56987015	T	C	-0.10552	0.012872	67.2	2.452E-16
SLC12A3	rs12923459	56989830	A	G	0.072051	0.0119187	36.54462	1.492E-09
SLC12A3	rs7203286	56986762	T	G	0.072502	0.0120419	36.250061	1.736E-09
SLC12A3	rs12149545	56993161	A	G	-0.10676	0.0128195	69.358448	8.208E-17
SLC12A3	rs56228609	56987765	T	C	-0.10851	0.0129754	69.939421	6.114E-17
SLC12A3	rs72786786	56985514	A	G	-0.10756	0.0133653	64.760749	8.457E-16
SLC12A3	rs711751	56993909	A	C	0.098694	0.0155733	40.162776	2.337E-10
ACE	rs9910703	60971504	A	G	-0.31157	0.0697489	19.953662	7.934E-06

ACE	rs61198867	61048042	T	C	-0.14498	0.0301907	23.061513	1.569E-06
ACE	rs56300715	61051972	T	G	-0.1454	0.0302051	23.170978	1.482E-06
ACE	rs56115484	61053966	T	C	-0.14406	0.0302073	22.744746	1.85E-06
ACE	rs62076587	61054474	C	A	-0.14225	0.0301504	22.260279	2.381E-06
ACE	rs4968639	61055390	G	A	-0.14432	0.0301722	22.87784	1.726E-06
ACE	rs9914354	61063555	G	C	-0.13717	0.0301957	20.63582	5.555E-06
ACE	rs9898603	61082410	C	T	-0.11732	0.0251465	21.766521	3.079E-06
ACE	rs1509822	61085984	T	A	-0.1175	0.0250892	21.933205	2.823E-06
ACE	rs5821379	61089917	C	G	-0.11875	0.0251213	22.346642	2.276E-06
ACE	rs112075512	61096710	G	T	-0.11875	0.0251213	22.346642	2.276E-06
ACE	rs12051630	61109643	A	G	-0.13685	0.0304989	20.133886	7.221E-06
ACE	rs4968751	61123287	T	C	-0.13823	0.0303501	20.743011	5.252E-06
ACE	rs11871269	61131801	T	C	-0.1375	0.0305153	20.301986	6.613E-06
ACE	rs59631404	61138449	T	C	-0.13685	0.0304989	20.133886	7.221E-06
ACE	rs143073010	61162836	A	T	-0.1375	0.0305153	20.301986	6.613E-06
ACE	rs6504150	61170174	G	A	-0.11187	0.0253176	19.524952	9.929E-06
ACE	rs7213524	61171554	G	A	-0.13746	0.0306173	20.157555	7.132E-06
ACE	rs11361218	61176223	G	GA	-0.14261	0.030188	22.318019	2.31E-06
ACE	rs62076633	61179440	C	T	-0.14432	0.0301722	22.87784	1.726E-06
ACE	rs6504152	61189826	G	A	-0.14393	0.0303011	22.561175	2.036E-06
ACE	rs11273482	61201133	G	C	-0.13535	0.0270665	25.007205	5.712E-07
ACE	rs9892875	61203431	A	C	-0.12129	0.0251331	23.290529	1.393E-06
ACE	rs7216611	61205709	A	G	-0.11544	0.0249572	21.394684	3.738E-06

ACE	rs7221301	61206239	A	G	-0.11728	0.0247255	22.4991	2.102E-06
ACE	rs2024228	61207365	A	G	-0.11799	0.0249513	22.360144	2.26E-06
ACE	rs4968753	61211021	T	G	-0.11514	0.0249077	21.370534	3.785E-06
ACE	rs17683221	61213287	A	G	-0.11514	0.0249077	21.370534	3.785E-06
ACE	rs7209469	61214699	G	A	-0.11569	0.0250195	21.382053	3.763E-06
ACE	rs4968754	61216509	T	C	-0.11681	0.0249069	21.993704	2.735E-06
ACE	rs73341776	61217569	G	A	-0.37277	0.0775385	23.11189	1.528E-06
ACE	rs9908459	61217911	C	T	-0.11569	0.0250195	21.382053	3.763E-06
ACE	rs4387652	61219955	A	G	-0.1147	0.0247937	21.402616	3.723E-06
ACE	rs28626208	61222954	T	C	-0.11514	0.0249077	21.370534	3.785E-06
ACE	rs73323712	61227391	C	T	-0.36203	0.0776966	21.710663	3.17E-06
ACE	rs17683399	61238199	G	A	-0.11483	0.0248348	21.377982	3.771E-06
ACE	rs9913027	61241107	T	C	-0.11444	0.0249082	21.108056	4.341E-06
ACE	rs17683477	61241793	T	C	-0.11444	0.0249082	21.108056	4.341E-06
ACE	rs9895619	61242897	A	G	-0.11444	0.0249082	21.108056	4.341E-06
ACE	rs1860660	61244803	G	A	-0.11471	0.0249732	21.100097	4.359E-06
ACE	rs4968755	61252252	G	A	-0.1114	0.0246441	20.43468	6.17E-06
ACE	rs1029883	61257359	G	T	-0.11261	0.0246306	20.903517	4.83E-06
ACE	rs79736985	61260434	C	G	-0.11052	0.0244854	20.374686	6.367E-06
ACE	rs78291167	61263355	A	T	-0.36203	0.0776966	21.710663	3.17E-06
ACE	rs73323736	61264765	C	T	-0.37277	0.0775385	23.11189	1.528E-06
ACE	rs2440140	61266803	A	C	-0.11418	0.0248627	21.091814	4.378E-06
ACE	rs2440141	61267003	A	G	-0.11317	0.0246346	21.105823	4.346E-06
ACE	rs8075731	61267502	G	C	-0.36203	0.0776966	21.710663	3.17E-06
ACE	rs2440142	61267567	G	A	-0.11445	0.0249265	21.081863	4.401E-06

ACE	rs2447455	61269838	A	G	-0.11418	0.0248627	21.091814	4.378E-06
ACE	rs2447454	61270972	T	A	-0.11418	0.0248627	21.091814	4.378E-06
ACE	rs10710409	61279187	A	T	-0.11418	0.0248627	21.091814	4.378E-06
ACE	rs7207314	61286621	A	G	-0.38912	0.0801039	23.597405	1.187E-06
ACE	rs1518774	61287445	G	T	-0.11038	0.0246674	20.023588	7.65E-06
ACE	rs8065101	61288366	G	C	-0.36203	0.0776966	21.710663	3.17E-06
ACE	rs2927291	61297892	A	G	-0.11176	0.0247909	20.321927	6.545E-06
ACE	rs2440137	61300010	C	T	-0.11038	0.0245981	20.137301	7.208E-06
ACE	rs1120106	61300328	T	C	-0.11038	0.0245981	20.137301	7.208E-06
ACE	rs73323776	61300353	T	C	-0.36111	0.0777072	21.594925	3.367E-06
ACE	rs10506520	61302469	G	C	-0.36203	0.0776966	21.710663	3.17E-06
ACE	rs8074720	61303051	T	A	-0.36203	0.0776966	21.710663	3.17E-06
ACE	rs16946811	61303694	T	C	-0.36185	0.0779003	21.576395	3.4E-06
ACE	rs112616618	61304947	A	G	-0.36111	0.0777072	21.594925	3.367E-06
ACE	rs2197262	61306491	A	T	-0.11418	0.0248627	21.091814	4.378E-06
ACE	rs2440146	61306995	C	T	-0.1157	0.0248923	21.604498	3.351E-06
ACE	rs991041	61311784	A	T	-0.11445	0.0249265	21.081863	4.401E-06
ACE	rs140876451	61313128	T	A	-0.36111	0.0777072	21.594925	3.367E-06
ACE	rs2460111	61315272	A	G	-0.11418	0.0248627	21.091814	4.378E-06
ACE	rs2447442	61318077	T	C	-0.11317	0.0246346	21.105823	4.346E-06
ACE	rs2460110	61319197	A	G	-0.1138	0.0249607	20.785273	5.138E-06
ACE	rs113392954	61323064	A	G	-0.36111	0.0777072	21.594925	3.367E-06
ACE	rs2460109	61323412	T	C	-0.11327	0.0246723	21.075968	4.414E-06
ACE	rs1518772	61326116	A	G	-0.11414	0.0248159	21.154758	4.236E-06

ACE	rs35406446	61327437	T	A	-0.11244	0.0245754	20.932317	4.758E-06
ACE	rs138850799	61330443	C	G	-0.36111	0.0777072	21.594925	3.367E-06
ACE	rs7223080	61331460	G	A	-0.37277	0.0775385	23.11189	1.528E-06
ACE	rs2120255	61335497	A	C	-0.11317	0.0246346	21.105823	4.346E-06
ACE	rs2447443	61337555	C	G	-0.11217	0.0246593	20.690392	5.399E-06
ACE	rs1518778	61347429	T	C	-0.36203	0.0783124	21.371512	3.784E-06
ACE	rs2920444	61350244	G	A	-0.11391	0.0249933	20.771184	5.176E-06
ACE	rs2460108	61361805	C	A	-0.1153	0.0248707	21.493404	3.55E-06
ACE	rs1899634	61362681	C	T	-0.11391	0.0249933	20.771184	5.176E-06
ACE	rs144797286	61367303	C	T	-0.37277	0.0775385	23.11189	1.528E-06
ACE	rs1596354	61375289	A	G	-0.12667	0.0267123	22.488081	2.114E-06
ACE	rs6504159	61382222	C	G	-0.38141	0.0776298	24.139315	8.961E-07
ACE	rs73325727	61383254	A	G	-0.36111	0.0777072	21.594925	3.367E-06
ACE	rs2447453	61383304	C	G	-0.11364	0.0249286	20.781011	5.149E-06
ACE	rs2907466	61386455	A	G	-0.11343	0.0249532	20.664204	5.473E-06
ACE	rs2447448	61386738	A	G	-0.11343	0.0249532	20.664204	5.473E-06
ACE	rs141306579	61386792	G	A	-0.36111	0.0777072	21.594925	3.367E-06
ACE	rs150165891	61387137	A	T	-0.36111	0.0777072	21.594925	3.367E-06
ACE	rs2429424	61389639	G	A	-0.11189	0.0247887	20.375398	6.364E-06
ACE	rs16715	61393656	G	A	-0.13117	0.0267563	24.033128	9.47E-07
ACE	rs147569695	61394313	G	C	-0.12642	0.026713	22.397523	2.217E-06
ACE	rs2447451	61397772	T	C	-0.11418	0.0248627	21.091814	4.378E-06
ACE	rs2447452	61398797	T	C	-0.11418	0.0248627	21.091814	4.378E-06

ACE	rs2447447	61400809	G	A	-0.11133	0.0250305	19.780914	8.684E-06
ACE	rs2429430	61403987	A	G	-0.11293	0.0248626	20.630886	5.569E-06
ACE	rs2429431	61411538	G	C	-0.11476	0.024836	21.351722	3.823E-06
ACE	rs2429432	61414403	A	G	-0.11453	0.0247759	21.36691	3.793E-06
ACE	rs3833118	61417820	T	A	-0.13041	0.0267664	23.737876	1.104E-06
ACE	rs1015760	61417882	A	C	-0.11547	0.0249174	21.473498	3.587E-06
ACE	rs3809721	61431754	T	G	-0.11318	0.0248897	20.67577	5.44E-06
ACE	rs4968765	61432774	G	A	-0.11302	0.0248038	20.762973	5.198E-06
ACE	rs4968642	61433121	A	G	-0.11291	0.0247633	20.78931	5.127E-06
ACE	rs7210570	61436852	G	A	-0.11302	0.0248038	20.762973	5.198E-06
ACE	rs4968769	61446962	T	C	-0.11122	0.0247338	20.219046	6.906E-06
ACE	rs2319812	61450418	G	C	-0.11317	0.0250649	20.385937	6.329E-06
ACE	rs1029764	61459067	A	C	-0.11061	0.0247927	19.904395	8.141E-06
ACE	rs9893857	61463748	T	G	-0.11039	0.0245216	20.263876	6.746E-06
ACE	rs4968644	61468052	C	G	-0.11103	0.0249585	19.791331	8.637E-06
ACE	rs2270133	61473325	A	C	-0.11362	0.0245613	21.39966	3.728E-06
ACE	rs16946908	61477071	C	T	-0.37277	0.0775385	23.11189	1.528E-06
ACE	rs7211831	61484505	G	C	-0.35294	0.0780801	20.43271	6.177E-06
ACE	rs7224147	61488308	T	G	-0.11415	0.0248263	21.140001	4.269E-06
ACE	rs9889828	61505500	T	C	-0.11285	0.0246255	20.999175	4.595E-06
ACE	rs4968773	61515258	A	G	-0.11139	0.0248024	20.168872	7.089E-06
ACE	rs4968775	61518766	C	G	-0.11675	0.0244231	22.851759	1.75E-06
ACE	rs60234517	61522084	A	G	-0.11944	0.0245317	23.705634	1.123E-06

ACE	rs2058203	61523004	G	C	-0.1212	0.0245055	24.461219	7.582E-07
ACE	rs7221613	61526663	T	C	-0.39445	0.0779854	25.582696	4.238E-07
ACE	rs4968647	61527677	C	T	-0.11475	0.0249881	21.086333	4.39E-06
ACE	rs8065717	61528041	C	G	-0.12294	0.024742	24.690158	6.733E-07
ACE	rs4968776	61529580	G	A	-0.12706	0.0256821	24.478068	7.516E-07
ACE	rs6504162	61537402	C	T	-0.28995	0.0652453	19.749665	8.828E-06
ACE	rs56245266	61546089	C	T	-0.64465	0.0776217	68.972336	9.986E-17
ACE	rs7213516	61551550	A	G	-0.65152	0.0905218	51.801994	6.139E-13
ACE	rs7214530	61551605	G	T	-0.61087	0.0784029	60.706638	6.625E-15
ACE	rs4290	61552128	T	C	-0.59885	0.0793606	56.940578	4.492E-14
ACE	rs7211207	61554913	C	G	-0.40366	0.0706637	32.631139	1.114E-08
ACE	rs4300	61557286	T	C	-0.63865	0.0788652	65.577935	5.586E-16
ACE	rs4304	61557939	T	C	-0.70041	0.106825	42.988906	5.506E-11
ACE	rs4316	61562309	T	C	-0.12199	0.0252801	23.285785	1.396E-06
ACE	rs4319	61562489	T	A	-0.11928	0.0254407	21.981398	2.753E-06
ACE	rs4320	61562553	A	G	-0.11875	0.0253549	21.934554	2.821E-06
ACE	rs4321	61562774	C	T	-0.12404	0.0253836	23.880592	1.025E-06
ACE	rs4323	61562954	T	C	-0.11816	0.0253807	21.674469	3.231E-06
ACE	rs4324	61563171	G	A	-0.12481	0.0253816	24.181412	8.768E-07
ACE	rs4326	61563242	A	G	-0.12481	0.0253816	24.181412	8.768E-07
ACE	rs4327	61563273	G	C	-0.12481	0.0253816	24.181412	8.768E-07
ACE	rs4328	61563413	C	T	-0.70041	0.106825	42.988906	5.506E-11

ACE	rs4329	61563458	G	A	-0.12481	0.0253816	24.181412	8.768E-07
ACE	rs4330	61563661	A	C	0.124597	0.0254276	24.010678	9.58E-07
ACE	rs4331	61564052	G	A	-0.12469	0.0253585	24.178539	8.78E-07
ACE	rs4332	61564281	C	T	-0.12469	0.0253585	24.178539	8.78E-07
ACE	rs4333	61564522	C	T	-0.11319	0.0255568	19.616711	9.464E-06
ACE	rs4334	61564940	C	A	-0.12469	0.0253585	24.178539	8.78E-07
ACE	rs4335	61565025	A	G	-0.1175	0.0255955	21.07515	4.416E-06
ACE	rs4336	61565131	A	T	-0.12469	0.0253585	24.178539	8.78E-07
ACE	rs4337	61565133	T	G	-0.12469	0.0253585	24.178539	8.78E-07
ACE	rs1987692	61565525	A	T	-0.12144	0.0253235	22.997637	1.622E-06
ACE	rs4344	61566724	A	G	-0.12399	0.0252665	24.080702	9.238E-07
ACE	rs4349	61569144	T	C	-0.54257	0.0790529	47.105101	6.728E-12
ACE	rs4353	61570422	G	A	-0.12141	0.0251073	23.384622	1.326E-06
ACE	rs4357	61571630	T	C	-0.52075	0.0769875	45.752671	1.342E-11
ACE	rs4359	61572343	C	T	-0.12893	0.0252544	26.061546	3.307E-07
ACE	rs4362	61573761	C	T	-0.11537	0.024945	21.391877	3.744E-06
ACE	rs4363	61574492	A	G	-0.12096	0.0250998	23.223553	1.442E-06
ACE	rs4364	61574662	A	C	-0.73402	0.109987	44.537636	2.495E-11
ACE	rs56060049	61576243	A	G	-0.39235	0.0702868	31.159372	2.377E-08
ACE	rs58495134	61576732	C	T	-0.62747	0.107329	34.177967	5.029E-09
ACE	rs139215165	61577756	A	T	-0.43395	0.0685197	40.109799	2.401E-10
ACE	rs73330068	61577847	A	G	-0.64661	0.108483	35.52691	2.515E-09
ACE	rs73994129	61586471	G	A	-0.37582	0.0829691	20.517076	5.91E-06
ACE	rs2727278	62016704	G	A	-0.28488	0.0641433	19.725057	8.942E-06
ACE	rs2727277	62016860	T	A	-0.28488	0.0641433	19.725057	8.942E-06

ACE	rs11270863	62016944	A	T	-0.28488	0.0641433	19.725057	8.942E-06

Supplementary Table S3 | SMR results of antihypertensive drug targets and systolic blood pressure in discovery cohort.

Gene	SMR association			HEIDI Test	
	OR	95%CI	P-Value	P-Value	Number of SNPs
ACE	1.767	[1.153, 2.707]	**0.009**	0.266	7
ADRB1	1.314	[1.056, 1.635]	**0.013**	0.363	20
ADRB2	1.774	[1.210, 2.600]	**0.003**	0.708	9
SLC12A3	2.350	[1.408, 3.921]	**0.001**	0.319	20

Abbreviations: 95% CI: 95% confidence interval; eQTL: expression quantitative trait loci; GWAS: genome-wide association study; HEIDI: heterogeneity in dependent instruments; OR: odds ratio; SE: standard error; SMR: summary-data-based Mendelian rondomization; SNP: single nucleotide polymorphisms.

Supplementary Table S4 | Sensitivity and power analyses of Summary-data-based Mendelian randomization analyses in discovery and validation cohorts.

Clear Cell Renal Cell Carcinoma	Gene	HEIDI Test		Power
		P-Value	Number of SNPs	
Discovery Cohort	ACE	0.717	8	0.960
	ADRB1	0.782	20	0.870
	ADRB2	0.964	9	0.890
	SLC12A3	0.400	20	0.910
Validation Cohort (Finland cohort)	ACE	0.866	8	0.940
	ADRB1	0.167	20	1.000
	ADRB2	0.021	8	0.850
	SLC12A3	0.251	20	0.870

Supplementary Table S5 | Phenotypes with the most significant associations for ADRB1.

Top *P-value* in gene	Phenotype
1.30E-15	Essential hypertension
1.30E-15	Hypertension
3.90E-08	Eustachian tube disorders
3.40E-07	Chronic venous insufficiency
7.00E-07	Hallux rigidus

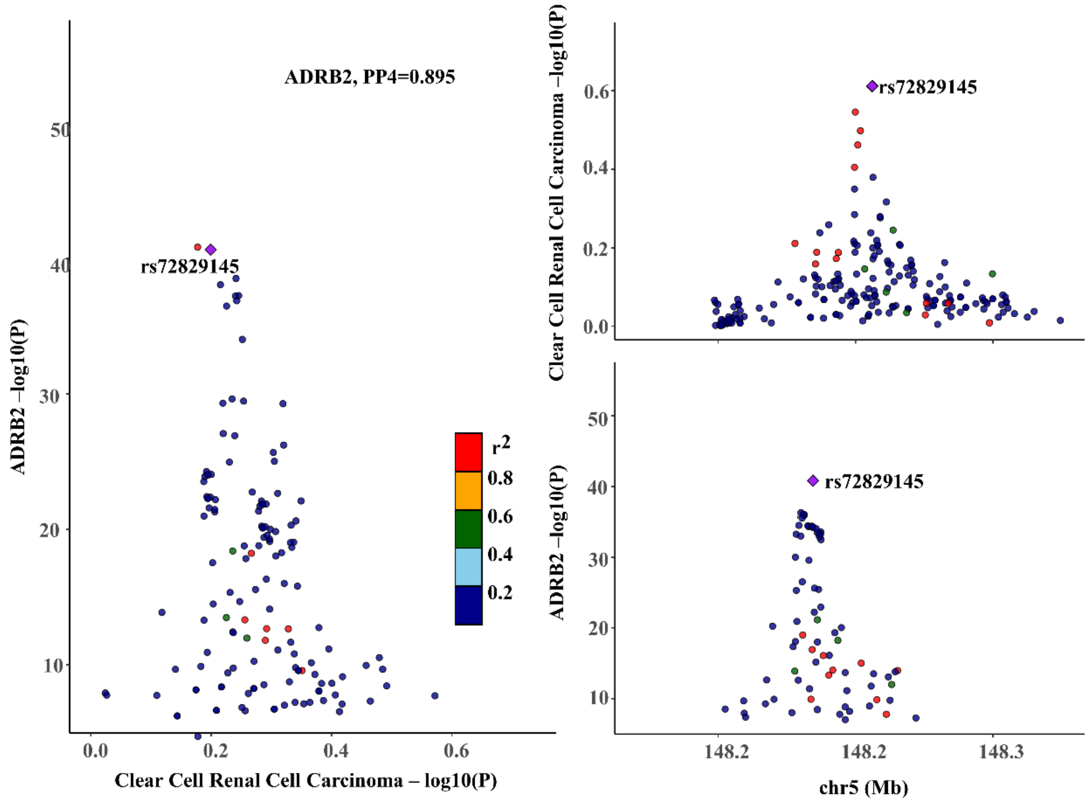

Supplementary Figure S1 | Regional Manhattan plot of associations of ADRB2 and risks of Clear Cell Renal Cell Carcinoma. The lead SNP is shown as a purple diamond. SNPs within ±500 kb of the antihypertensive drug target trait locus were included; p12=1×10−5, prior probability a SNP is associated with both ADRB2 and Clear Cell Renal Cell Carcinoma.

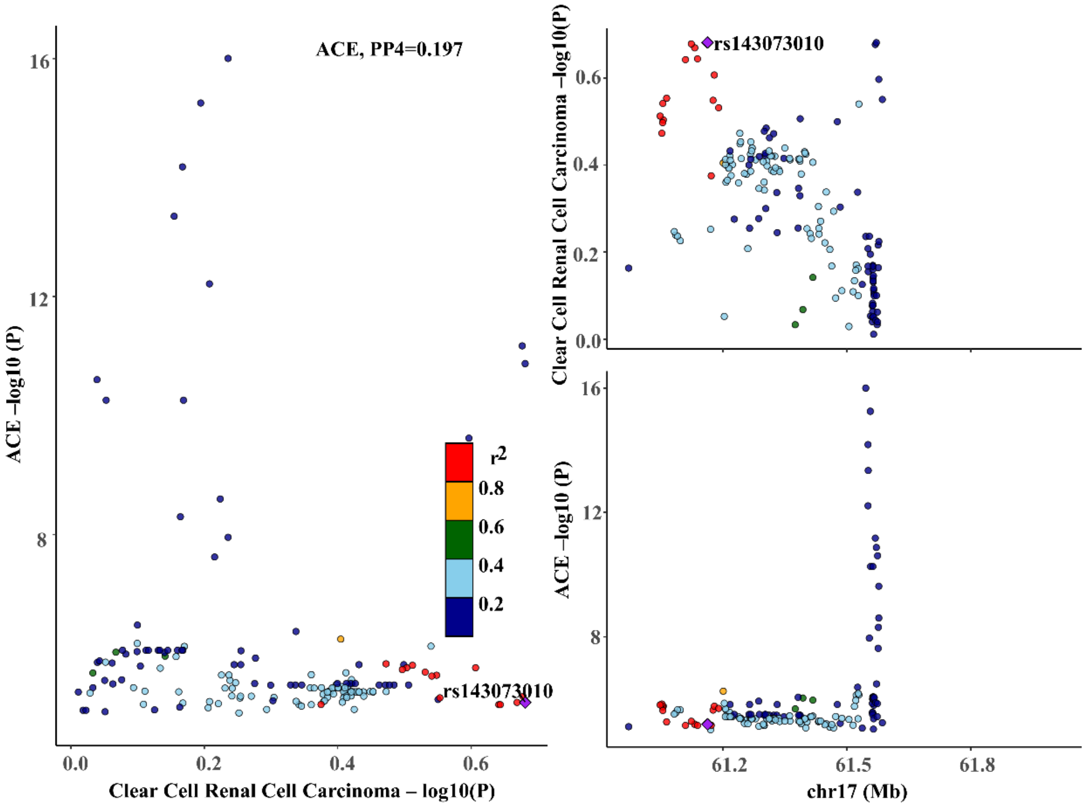

Supplementary Figure S2 | Regional Manhattan plot of associations of ACE and risks of Clear Cell Renal Cell Carcinoma. The lead SNP is shown as a purple diamond. SNPs within ±500 kb of the antihypertensive drug target trait locus were included; p12=1×10−5, prior probability a SNP is associated with both ACE and Clear Cell Renal Cell Carcinoma.

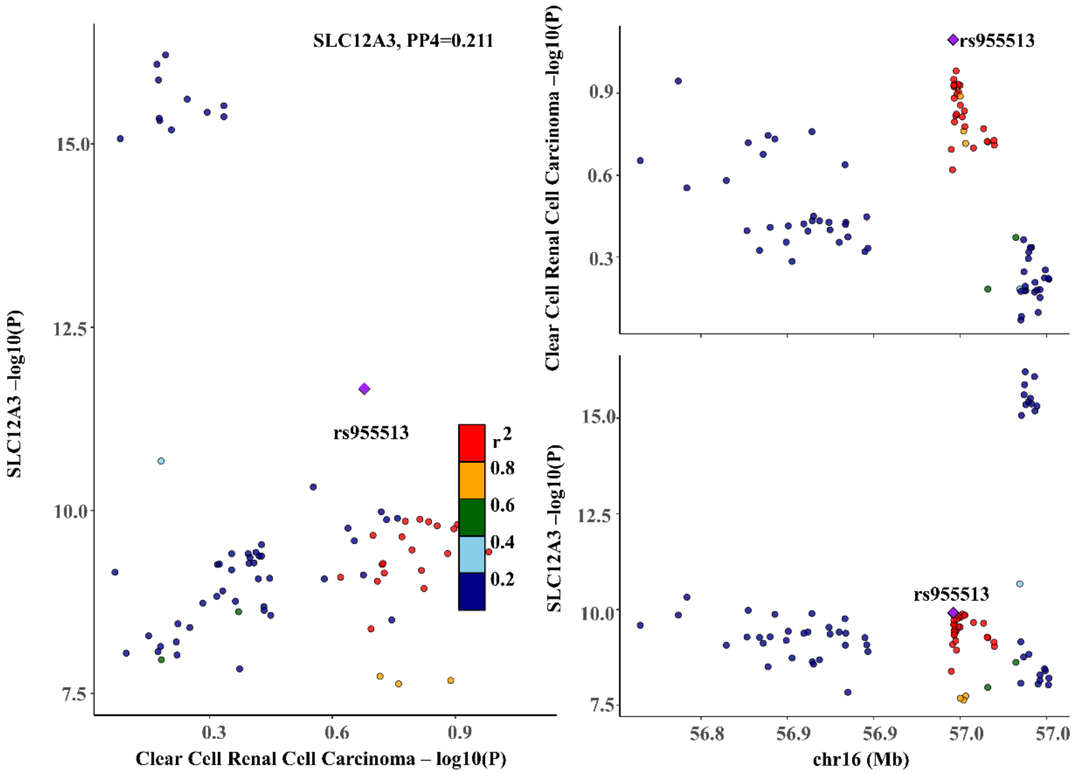

Supplementary Figure S3 | Regional Manhattan plot of associations of SLC12A3 and risks of Clear Cell Renal Cell Carcinoma. The lead SNP is shown as a purple diamond. SNPs within ±500 kb of the antihypertensive drug target trait locus were included; p12=1×10−5, prior probability a SNP is associated with both SLC12A3 and Clear Cell Renal Cell Carcinoma.
